# Supplementary material for: Suppression of kernel vibrations by layer-by-layer ligand engineering boosts photoluminescence efficiency of gold nanoclusters
Source: Nat Commun. 2023 Feb 7;14:658. doi: 10.1038/s41467-023-36387-2 (PMC9902523; doi:10.1038/s41467-023-36387-2)
Supplement: Supplementary file 1 — Supplementary Information [file 41467_2023_36387_MOESM1_ESM.pdf]

Supplementary Information

Suppression of kernel vibrations by layer-by-layer ligand engineering boosts photoluminescence efficiency of gold nanoclusters

Yuan Zhong,[1⊥] *Jiangwei Zhang,*[2⊥] *Tingting Li,*[3] *Wenwu Xu,*[4] *Qiaofeng Yao,*[5] *Min Lu,*[1] *Xue Bai,*[1] *Zhennan Wu,*[1*] *Jianping Xie,*[6*] *and Yu Zhang*[1*]

[1]State Key Laboratory of Integrated Optoelectronics, College of Electronic Science and Engineering, Jilin University, Changchun 130012, P. R. China

[2]Innovation Center of Energy Material and Chemistry; College of Chemistry and Chemical Engineering, Inner Mongolia University, Hohhot 010021, P. R. China

[3]College of Materials Science and Engineering, Jilin Jianzhu University, Changchun 130012, P. R. China

[4]Department of Physics, School of Physical Science and Technology, Ningbo University, Ningbo 315211, P. R. China

[5]Joint School of National University of Singapore and Tianjin University, International Campus of Tianjin University, Binhai New City, Fuzhou 350207, P. R. China

[6]Department of Chemical and Biomolecular Engineering, National University of Singapore, Singapore 117585, Singapore

[⊥]These authors contributed equally.

*Corresponding author. Email: wuzn@jlu.edu.cn; chexiej@nus.edu.sg; yuzhang@jlu.edu.cn

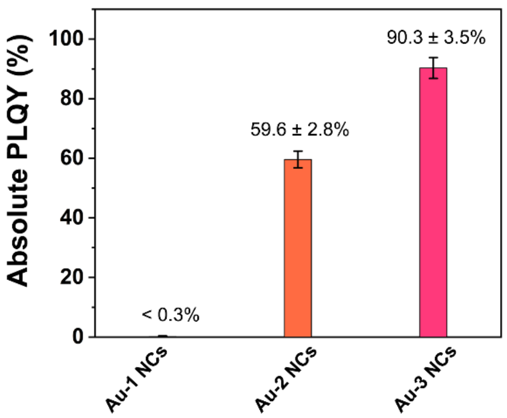

Supplementary Figure 1 The statistics of recorded PLQY values of < 0.3% for Au-1 NCs, 59.6 ± 2.8% for Au-2 NCs, and 90.3 ± 3.5% for Au-3 NCs, respectively. The absolute PLQY values were directly measured on a FLS1000 spectrofluorometer attached with an integrating sphere.

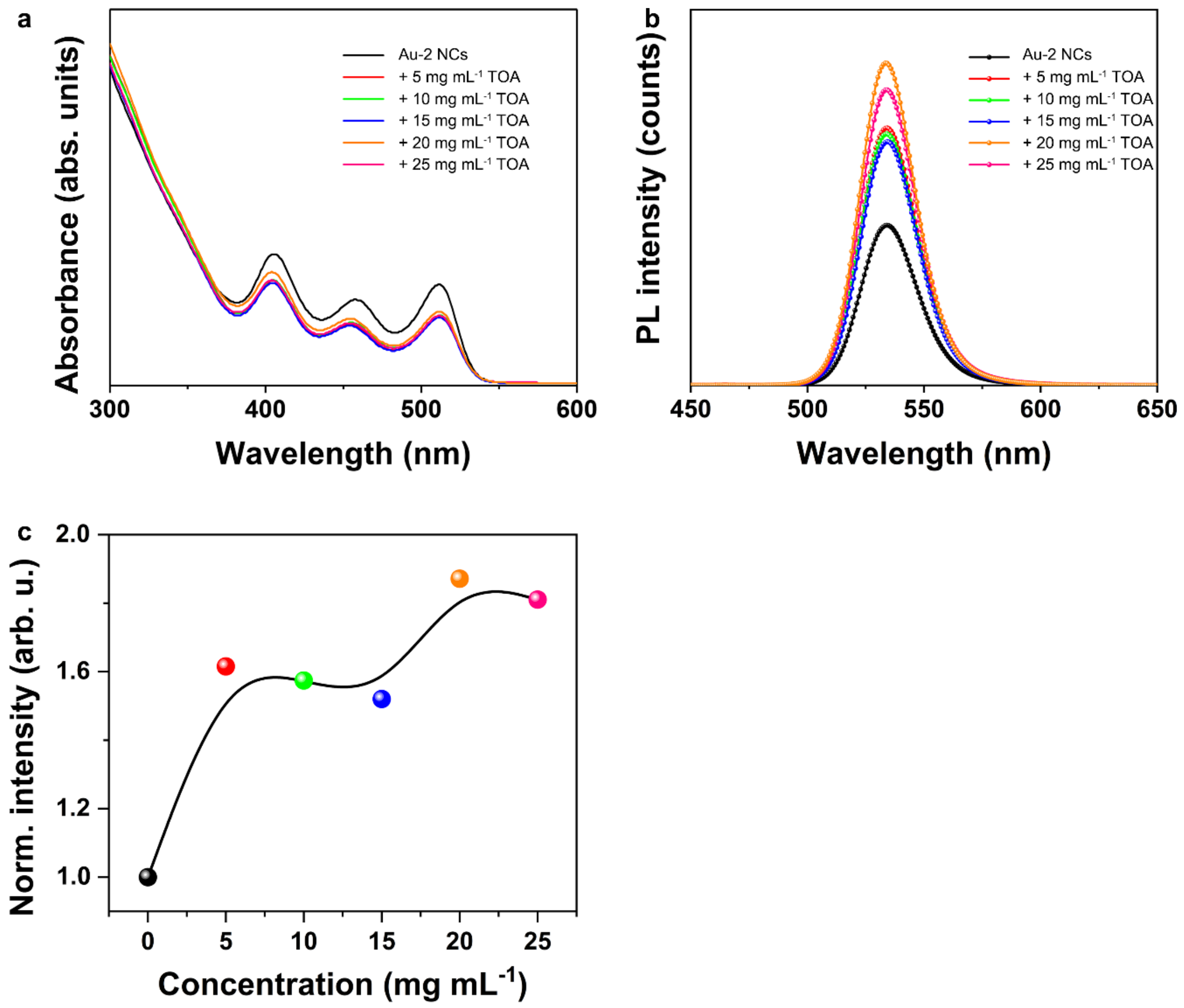

Supplementary Figure 2 Optical properties of Au-2 NCs added with different amount of TOA. TOA concentration-dependent (a) UV-vis absorbance and (b) PL spectra of Au-2 NCs added with different amount of TOA ligand. (c) The variation tendency of integrated emission intensity as a function of adding amount of TOA ligand in Au-2 NCs.

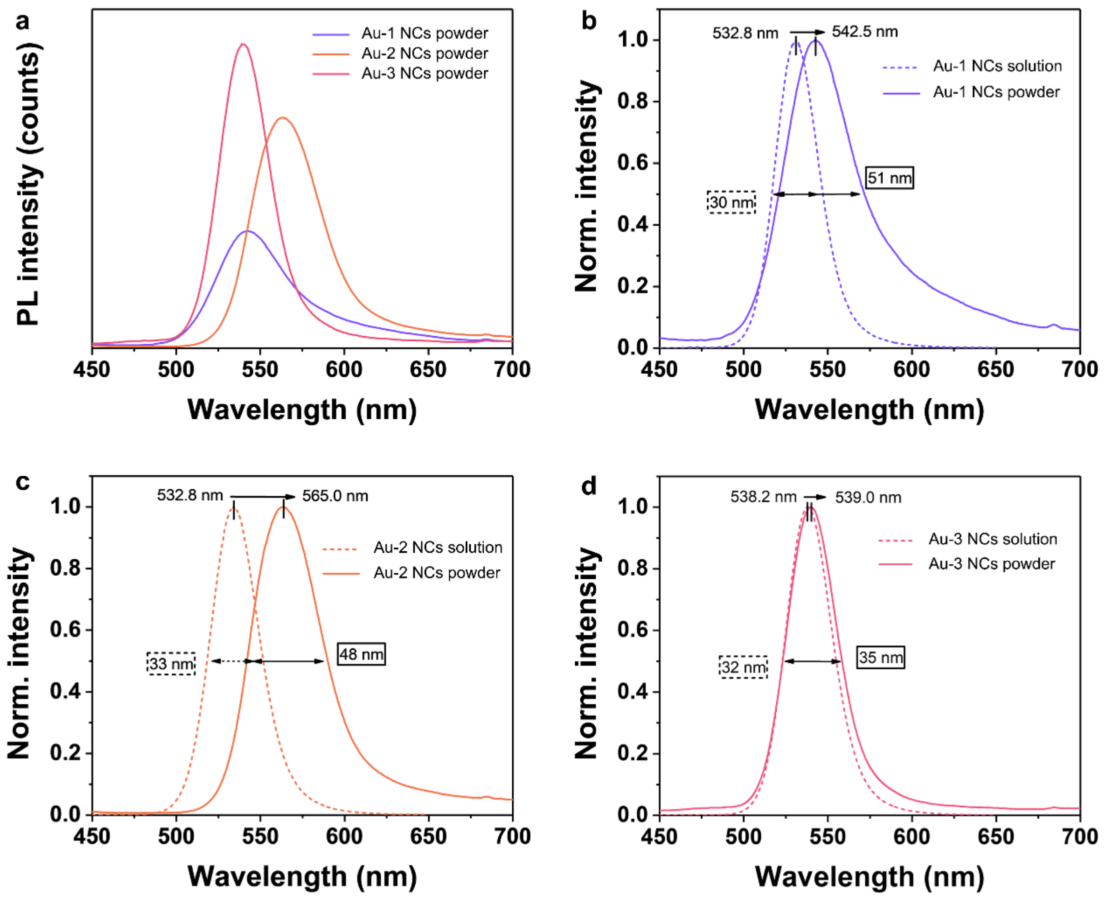

Supplementary Figure 3 Comparsion of emission spectra of Au-1, Au-2, and Au-3 NCs in solution and powder states. (a) Emission spectra of Au-1, Au-2, and Au-3 powders. The comparisons of normalized emission spectra in solution and powder state for (b) Au-1, (c) Au-2, (d) Au-3 NCs. For all three gold NCs, the emission patterns were red-shifted to different extents. This result is unambiguously distinct from classical AIE-type NCs, in which the lengthening of average metal(I)···metal(I) distance in the aggregated state would lead to the distinct blue shift in corresponding emission spectra. More especially, in compassion with Au-1 and Au-2 NCs, both the variations in peak position and FWHM of emission band are minimum for Au-3 NCs, revealing that the significantly rigidified surface in Au-3 NCs is a benefit to stabilize their PL properties.

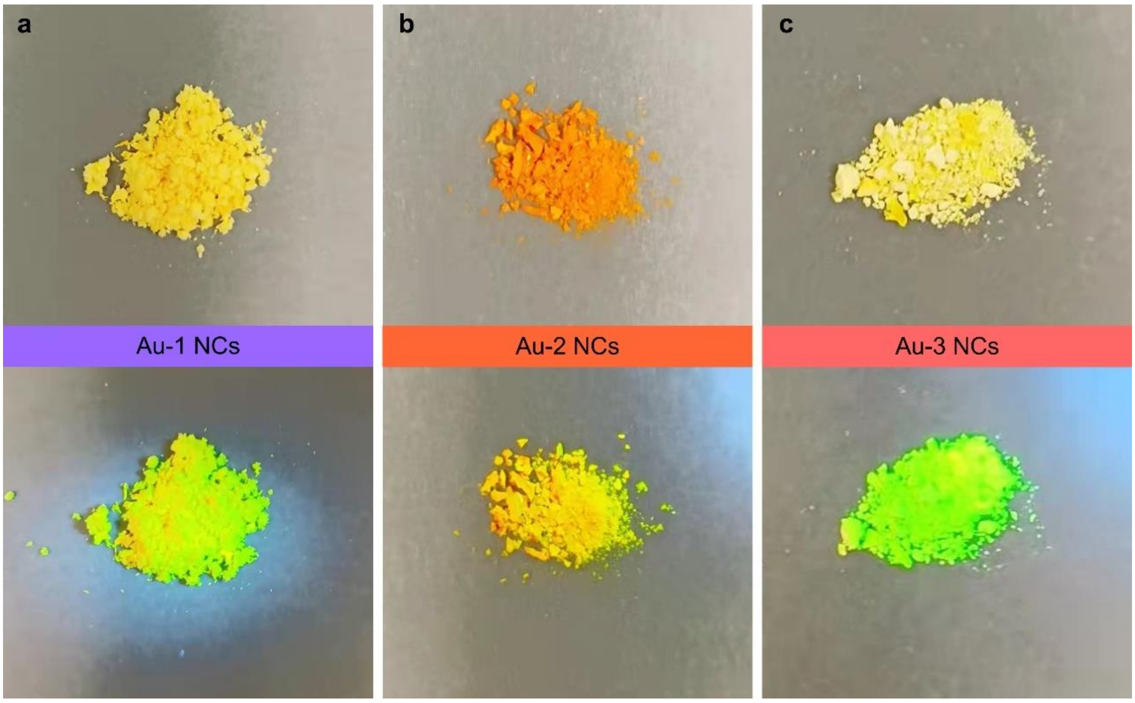

Supplementary Figure 4 Digital optical photographs of the powders of serial gold NCs. (a) Au-1, (b) Au-2, (c) Au-3 NCs. These photographs were taken under ambient atmosphere (top plane) and 365 nm UV light radiation (bottom plane).

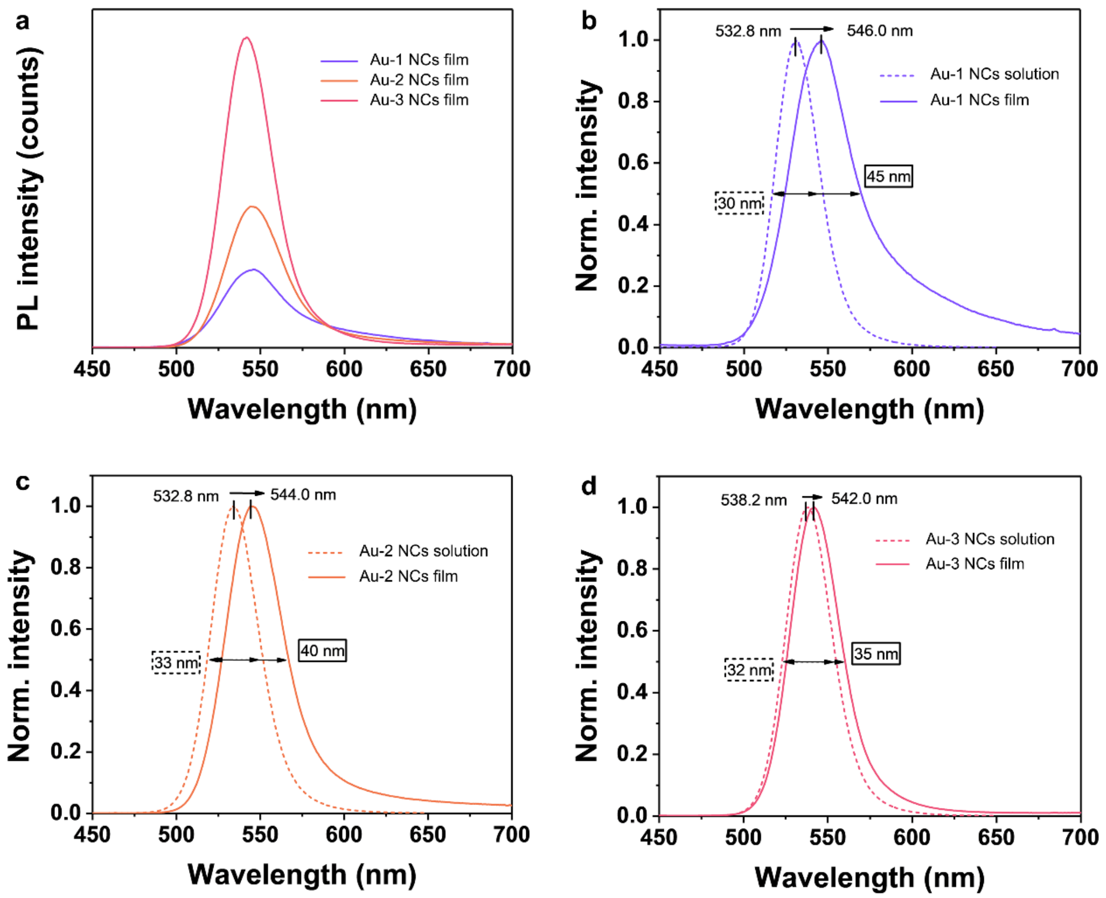

Supplementary Figure 5 Comparsion of emission spectra of Au-1, Au-2, and Au-3 NCs in solution and film states. (a) Emission spectra of Au-1, Au-2, and Au-3 films. The comparisons of normalized emission spectra in solution and film state: (b) Au-1, (c) Au-2, (d) Au-3 NCs.

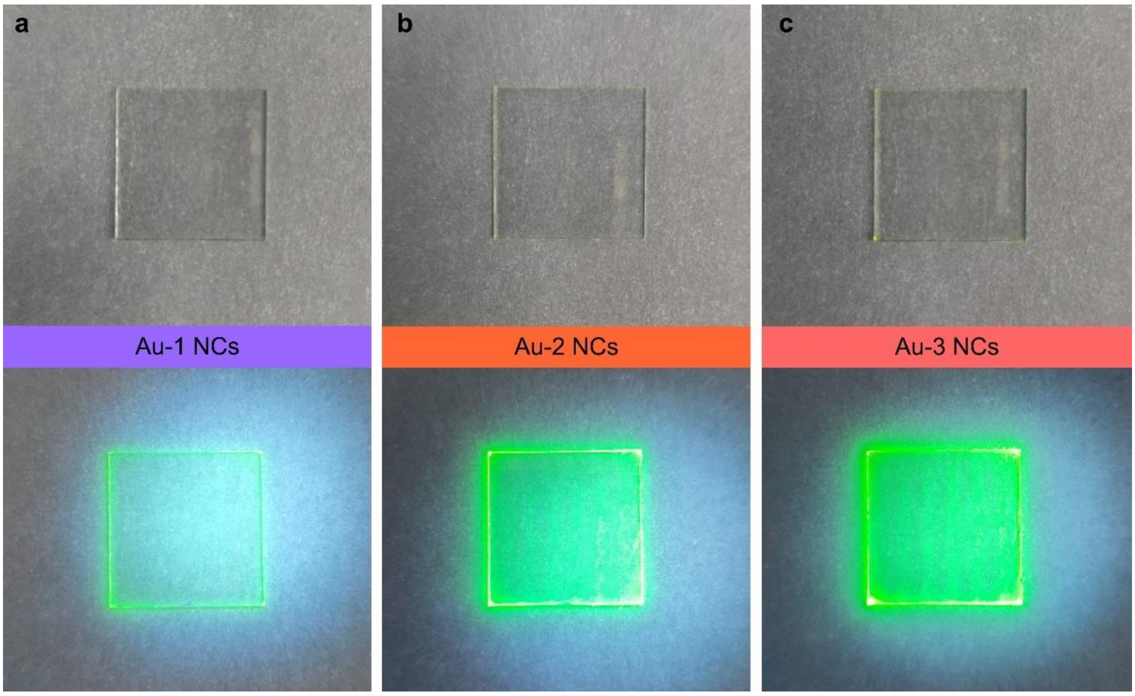

Supplementary Figure 6 Digital optical photographs of the powders of serial gold NCs. (a) Au-1, (b) Au-2, (c) Au-3 NCs. These photographs were taken under ambient atmosphere (top plane) and 365 nm near-ultraviolet (n-UV) light radiation (bottom plane).

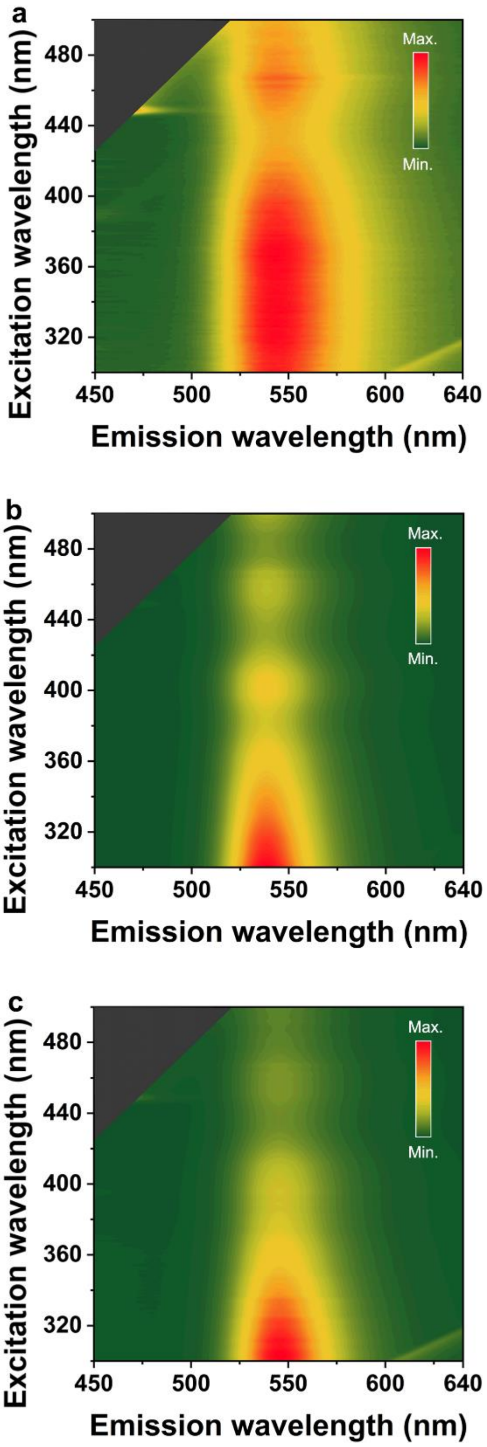

Supplementary Figure 7 2D excitation-emission contour mapping of serial gold NCs. (a) Au-1, (b) Au-2, and (c) Au-3 NCs. The horizontal and vertical dot lines refer to the excitation and emission peak positions, respectively.

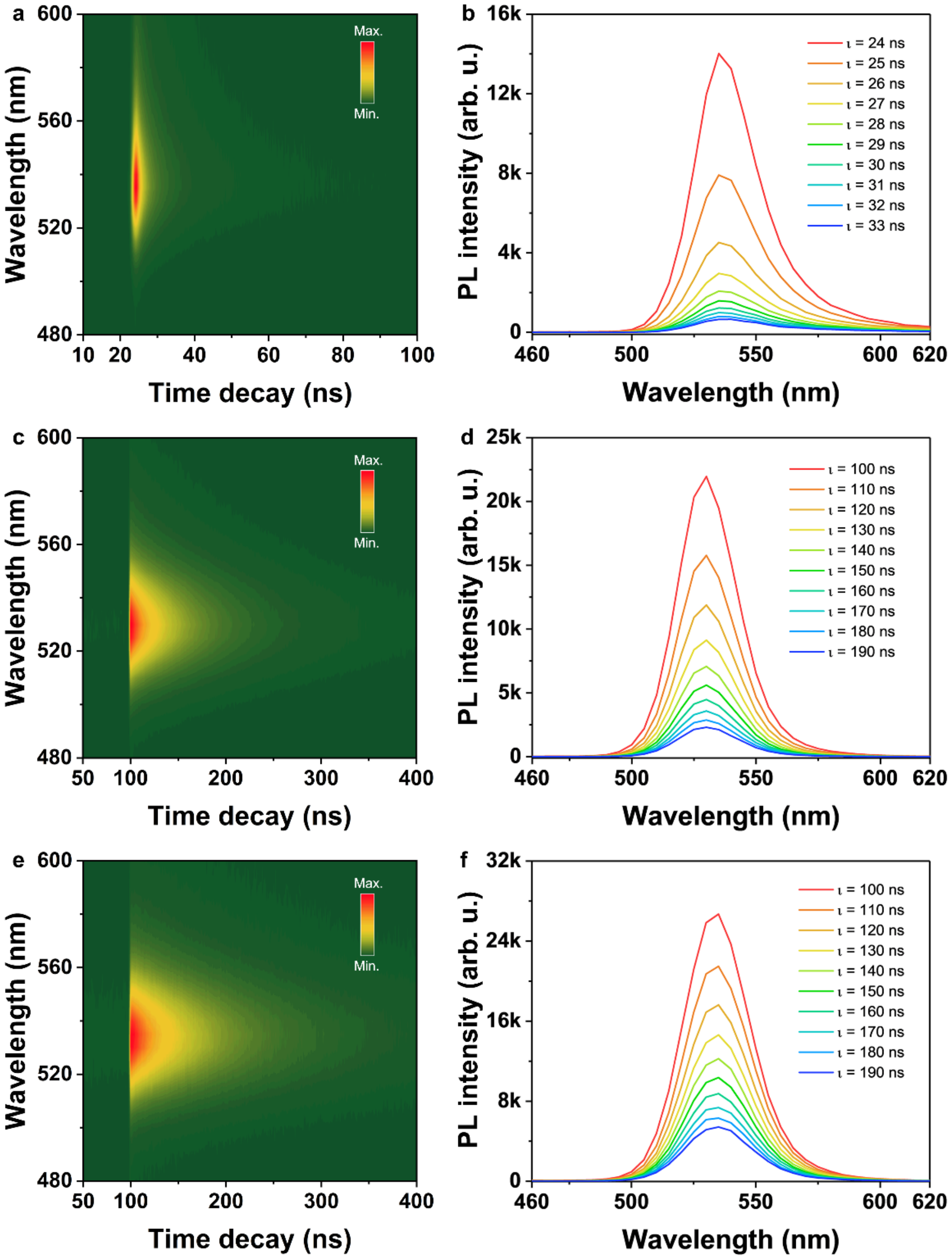

Supplementary Figure 8 Time-resolved optical properties of serial gold NCs. Time-resolved emission spectra (TRES) and selected emission spectra at different decay times of (a, b) Au-1 NCs, (c, d) Au-2 NCs, and (e, f) Au-3 NCs upon 405 nm excitation.

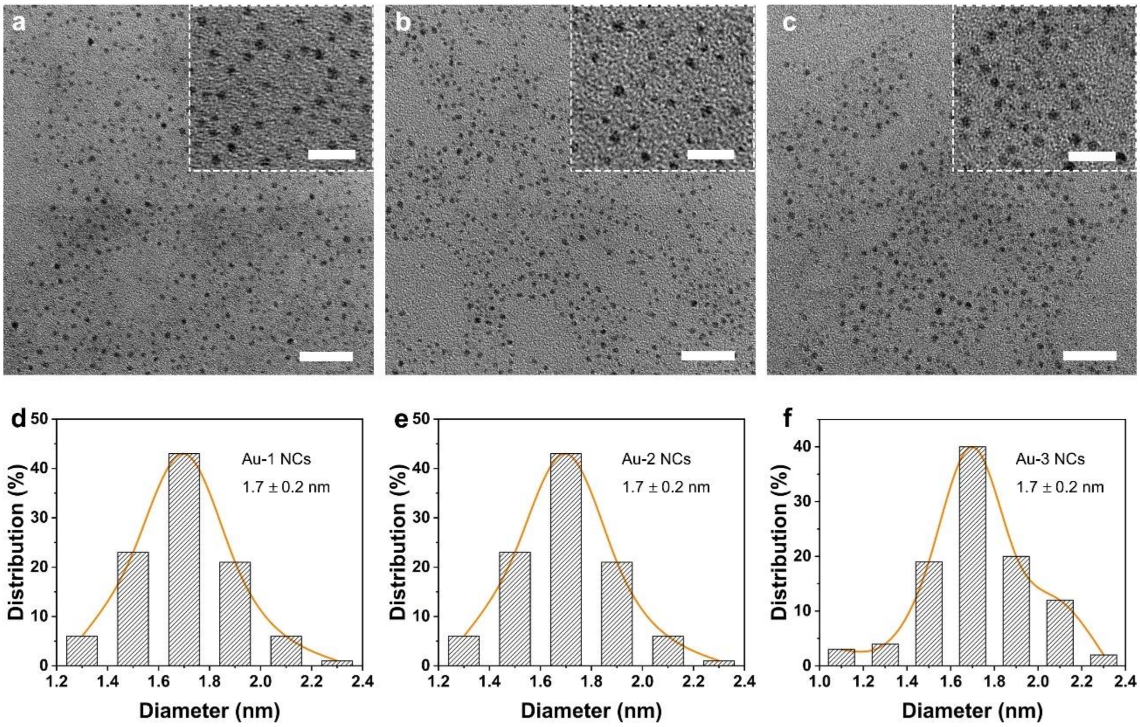

Supplementary Figure 9 TEM images and size statistics of as-synthesized (a, d) Au-1, (b, e) Au-2, and (c, f) Au-3 NCs. The scale bars are 20 nm in (a-c) and 10 nm in insets, respectively. The size data were collected from corresponding TEM images and a total of 100 NCs were measured for each image. The comparable size distribution reveals the gold core do not vary much after anchored with the second ARG and third TOA ligands.

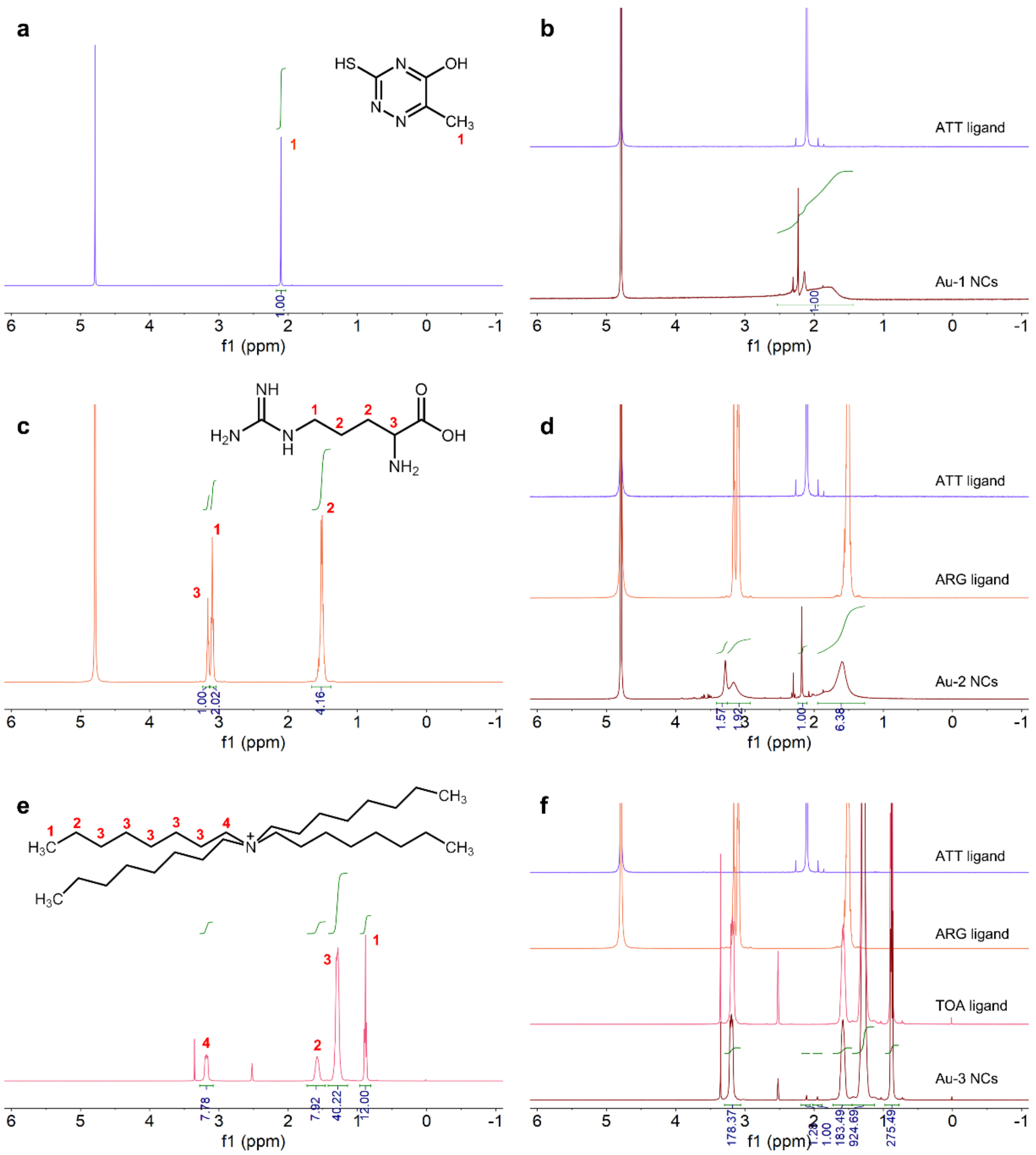

Supplementary Figure 10 ^{1}H-NMR spectra of serial gold NCs. (a) ATT ligand, (b) Au-1 NCs, (c) ARG ligand, (d) Au-2 NCs, (e) TOA ligand, and (f) Au-3 NCs. The sharp peak located at 4.79 ppm corresponds to the solvent residual signal of deuterium oxide (D_2O). Noting that the ^{1}H signals from the ATT ligand are relatively weak especially in the ^{1}H-NMR spectrum of Au-3 NCs. This result may be caused by the enhanced rigidity of surface ligands, which is capable of greatly suppressing the motion of protons in the innermost ligand layer of ATT. Moreover, after incorporating with TOA ligand, the as-generated electrostatic interaction between the terminal carboxyl of ARG and QACs of TOA can reduce the electron cloud density (ECD) around ARG ligand. This further results in the weakened shielding effect of ARG ligand, leading to the boosted chemical shift of protons in ARG towards the low field. Therefore, the ^{1}H signals of ARG ligand in Au-3 NC are superimposed with the intrinsic ^{1}H signals from TOA ligand at 1.58 and 3.20 ppm.

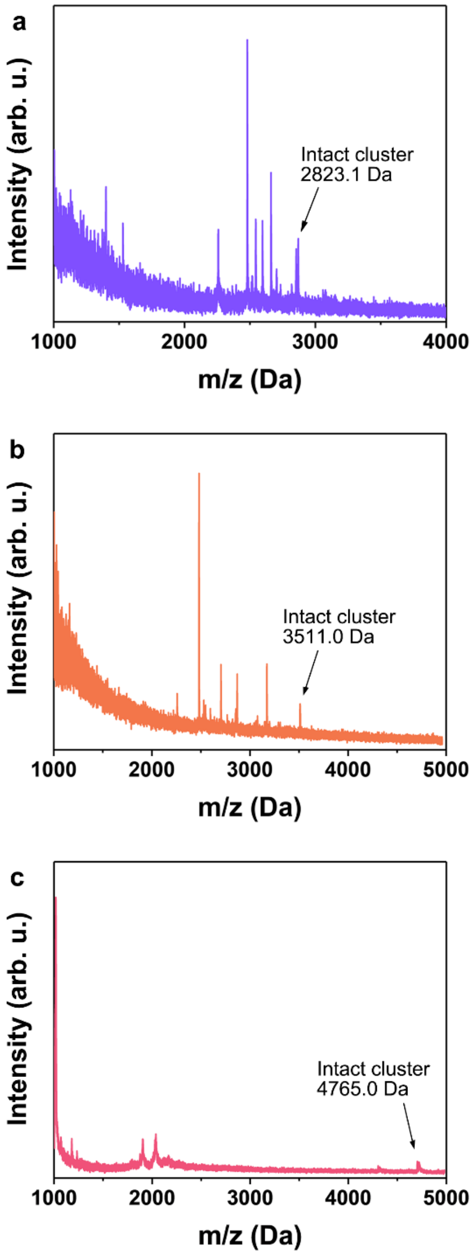

Supplementary Figure 11 Positive MALDI-TOF mass spectra of serial gold NCs. (a) Au-1, (b) Au-2, and (c) Au-3 NCs. DCTB was used as the matrix for all samples. The maximally prominent peaks were observed at 2823.1, 3511.0, and 4765.0 Da in (a), (b), and (c), respectively. There is a discrepancy of 22 Da between the experimental and theoretical mass values of Au-3 NCs. This can be caused by the ion exchange of one H^+ for one Na^+, whose corresponding *m*/*z* value increase with an interval of 22 Da [22 = 23(m_{Na+}) – 1(m_{H+})]. Other peaks in the mass spectrum with smaller mass values should originate from laser-induced fragmentations, even though DCTB matrix and very low laser pulse intensity were used throughout the measurements.

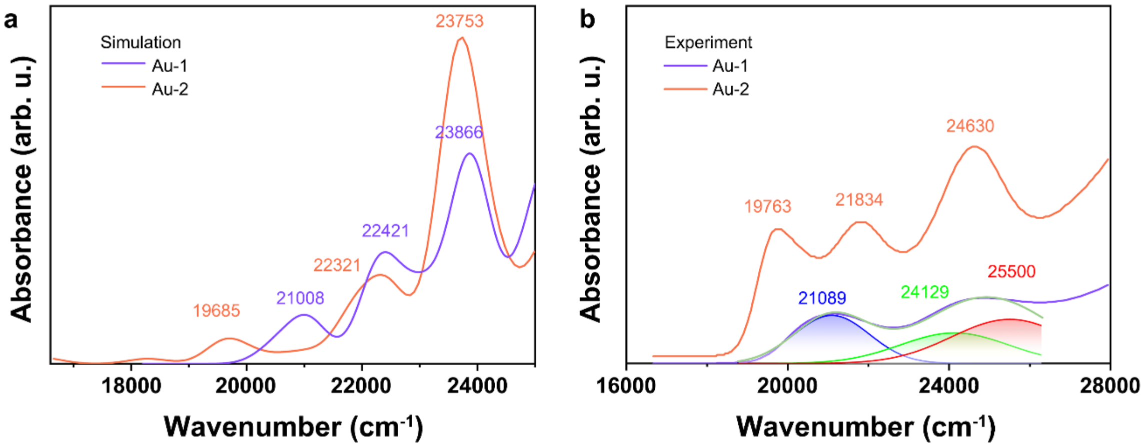

Supplementary Figure 12 Comparsion of simulated and experimental absorption of Au-1 and Au-2 NCs. (a) The calculated UV-vis absorption spectra of Au-1 and Au-2 NCs based on DFT simulation. (b) The experimental UV-vis absorption spectra of Au-1 and Au-2 NCs. The latter peak at 24875 cm^{-1} in experimental UV-vis absorption spectra of Au-1 NCs was fitted to be additional peaks at 24129 and 25500 cm^{-1}, which were colored in green and blue, respectively. Note that a discrepancy between the simulated absorption band and the experimental absorption profile exists. We attribute this slight difference to the implicit solvent model in our simulations. The implicit solvent model does not specifically describe the specific structure and distribution of solvent molecules near the solute, but simply considers the solvent environment as a polarizable continuum. The advantage of considering the solvent effect is that the average effect of the solvent can be represented without the need to consider the arrangement of various possible solvent layer molecules like the explicit solvent model, and it will not increase the computational time. Therefore, the implicit solvent model is widely used in the fields of quantum chemistry and molecular simulation. However, the disadvantage of the implicit solvent model is that it cannot represent strong interactions between solvent and solute, such as hydrogen bonds. Certain electronic excitations may involve charge transfer between solute and solvent, which obviously cannot be properly represented by the implicit solvent model.

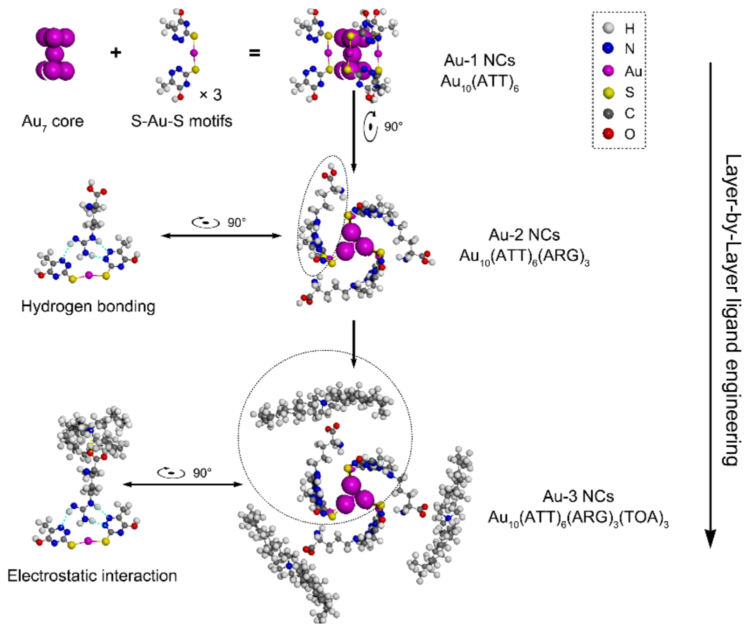

Supplementary Figure 13 The proposed structural evolution from Au-1 to Au-2 to Au-3 NCs through layer-by-layer triple-ligand self-assembly. In previous work finished by Pei et al.[1], they combined the genetic algorithm by using the local minimums primarily searched by the basin-hopping as the initial individuals in the search of local minima of $Au_{10}(SR)_6$. A genetic algorithm is an evolutionary algorithm based on the population, which generates new individuals by applying the crossover and mutation operation to the parent populations and achieves a better offspring according to the "survival of the fitness" principle. With the combination of the basin-hopping algorithm and genetic algorithm, they picked out the global minimum and some energetically degenerate or higher-lying metastable isomers, in which three stable isomers of $Au_{10}(SR)_6$ were identified. On this base, the most stable isomer with the lowest formation energies was employed to reconstruct the structures of Au-1, Au-2, and Au-3 NCs, and further simulate their corresponding absorption spectra.

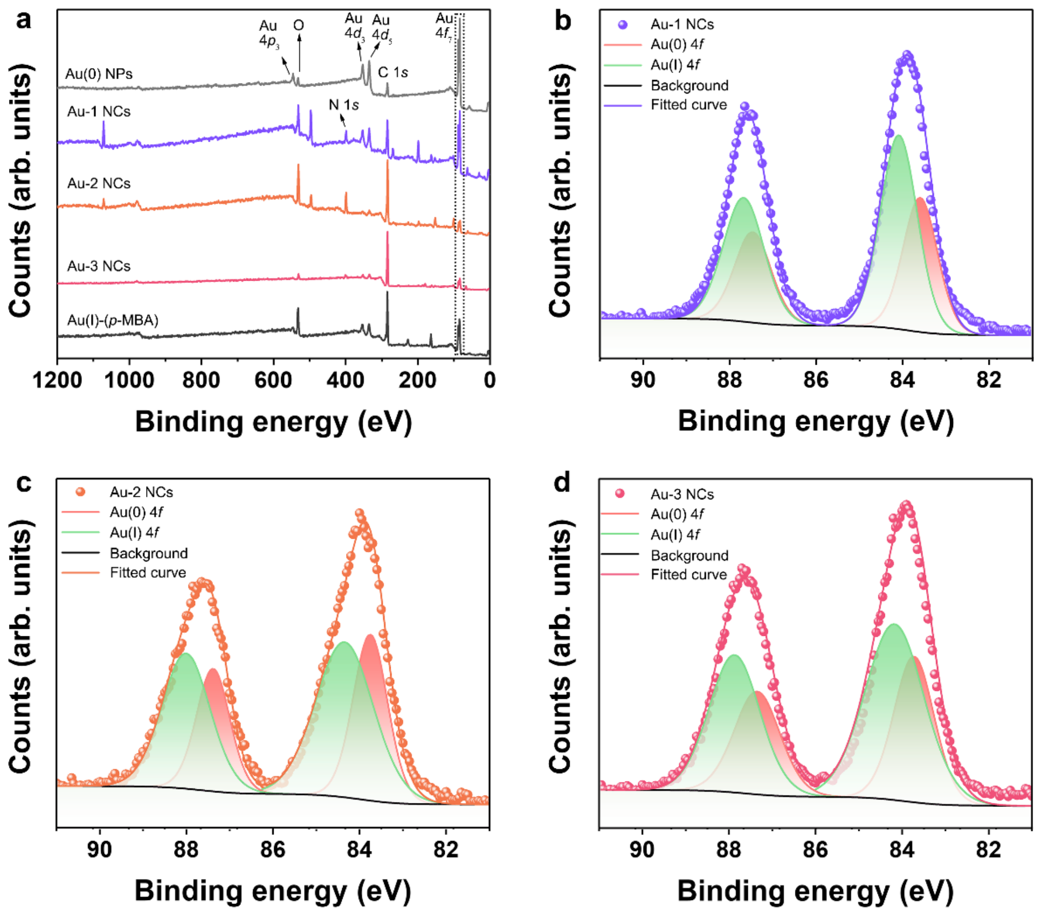

Supplementary Figure 14 XPS spectra of serial gold NCs. (a) XPS survey and Au 4*f* peak fitting of (b) Au-1, (c) Au-2, and (d) Au-3 NCs. The N element in the XPS surveys comes from the ATT ligand for Au-1 and Au-2 NCs, and comes both from ATT and TOA ligands for Au-3 NCs.

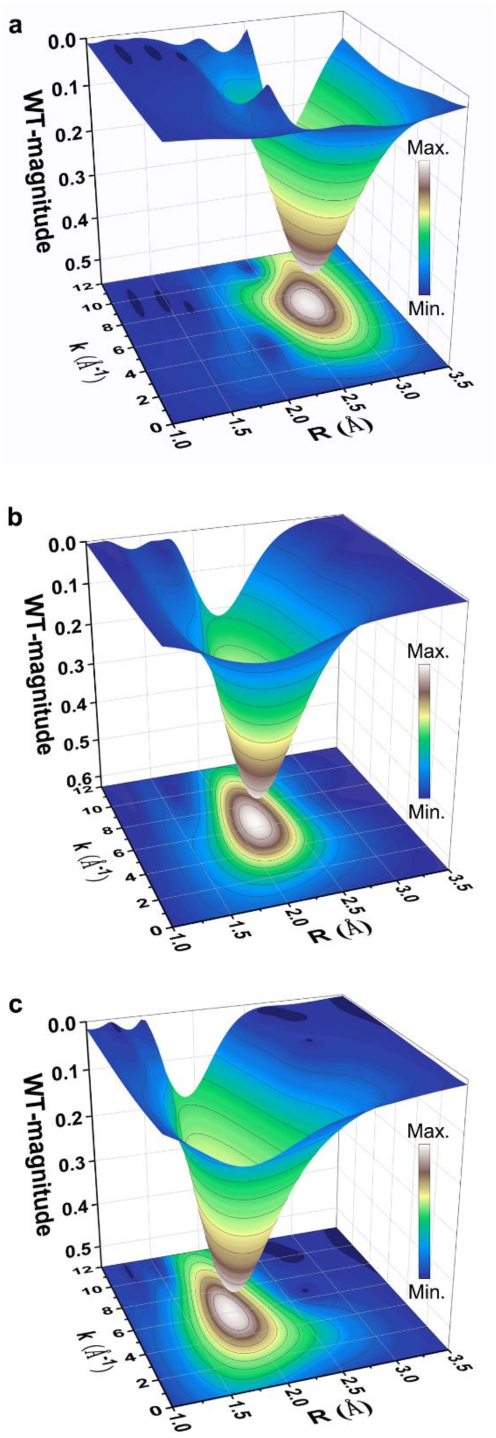

Supplementary Figure 15 3D WT for the k^2-weighted EXAFS signals of references. (a) Au foil, (b) AuS, and (c) Au_2O_3 references. The scattering path signals of Au-Au, Au-S, and Au-O bonding are peaking at [6.9, 2.75], [6.5, 2.18], and [6.1, 1.91], respectively.

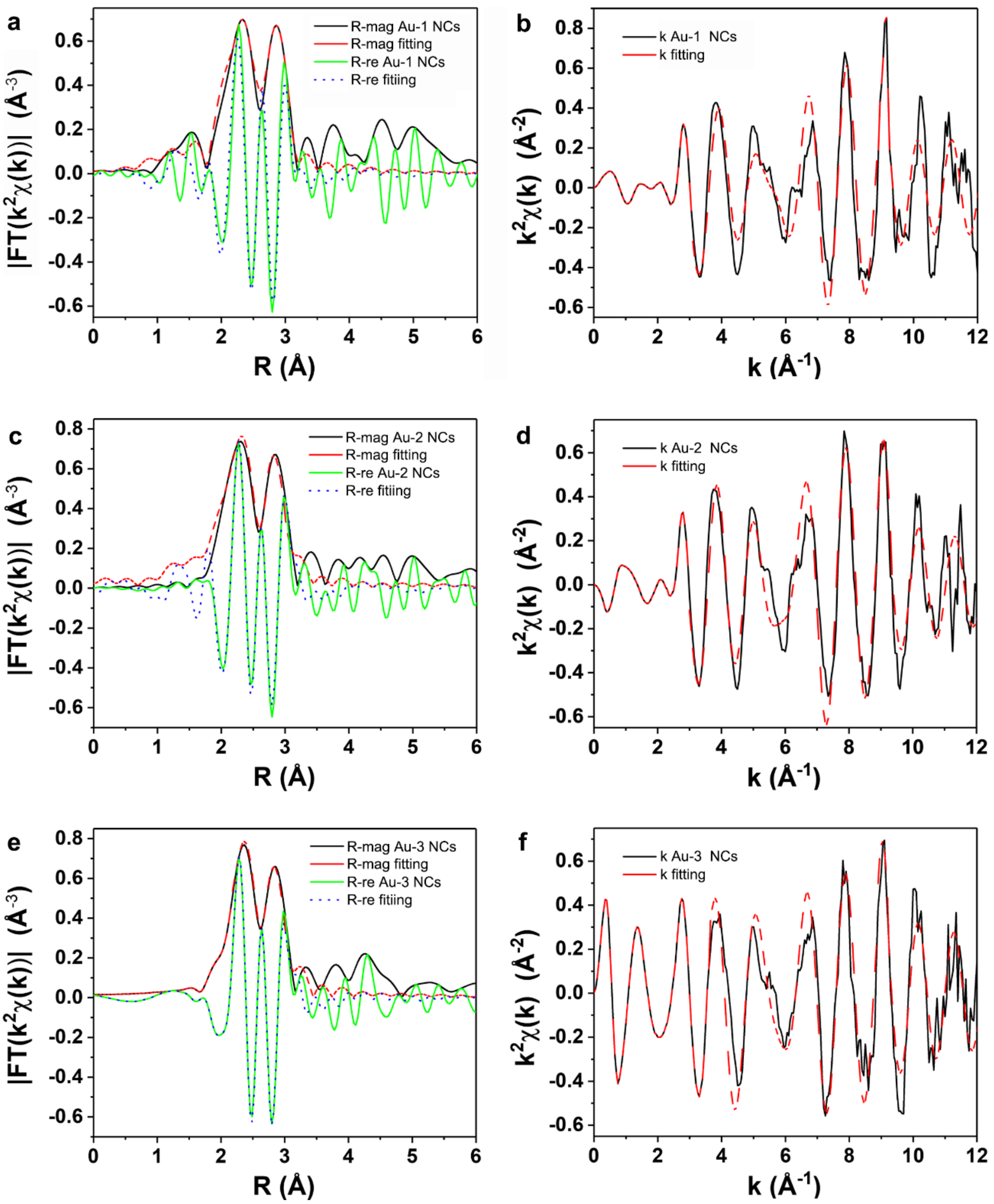

Supplementary Figure 16 FT of the k^2-weighted EXAFS spectra and their corresponding fitting in *R* space of (a) Au-1, (c) Au-2, and (e) Au-3 NCs with the magnitude and real component. $k^2\chi(k)$ space spectra and their corresponding fitting of (b) Au-1, (d) Au-2, and (f) Au-3 NCs.

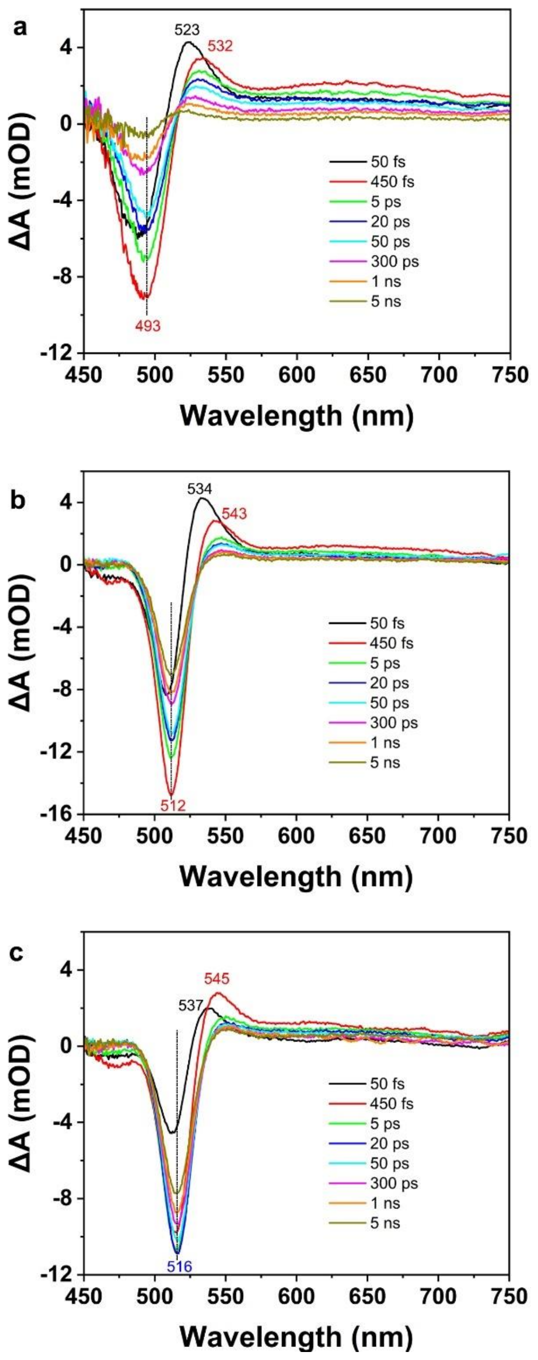

Supplementary Figure 17 Line-by-line TA spectra of serial gold NCs. TA spectra as a function of selected time delays from 50 fs to 5 ns for (a) Au-1 NCs, (b) Au-2 NCs, and (c) Au-3 NCs.

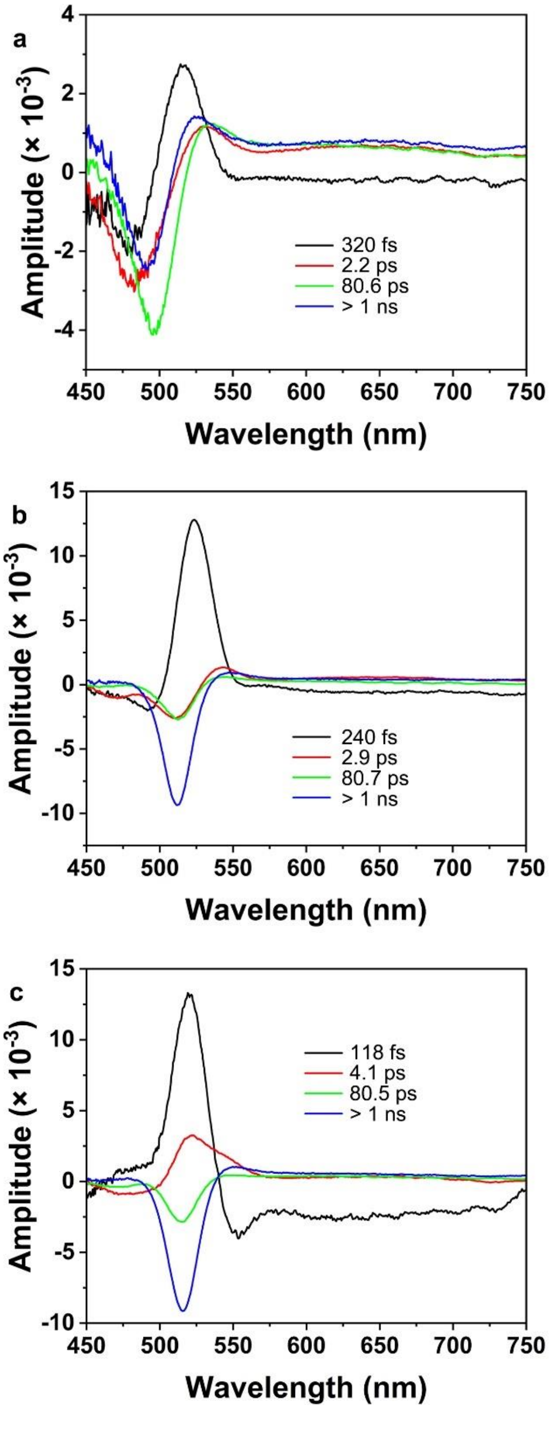

Supplementary Figure 18 Decay-associated spectra (DAS) of serial gold NCs. (a) Au-1, (b) Au-2, and (c) Au-3 NCs. These data were obtained from global fitting on their corresponding TA data.

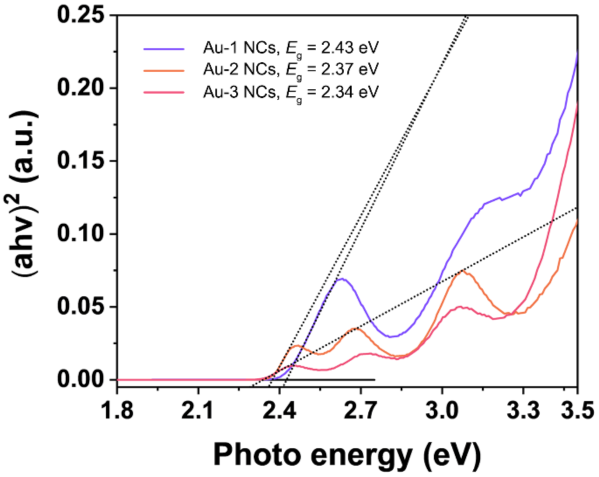

Supplementary Figure 19 Calculation of bandgap energies of Au-1, Au-2, and Au-3 NCs.

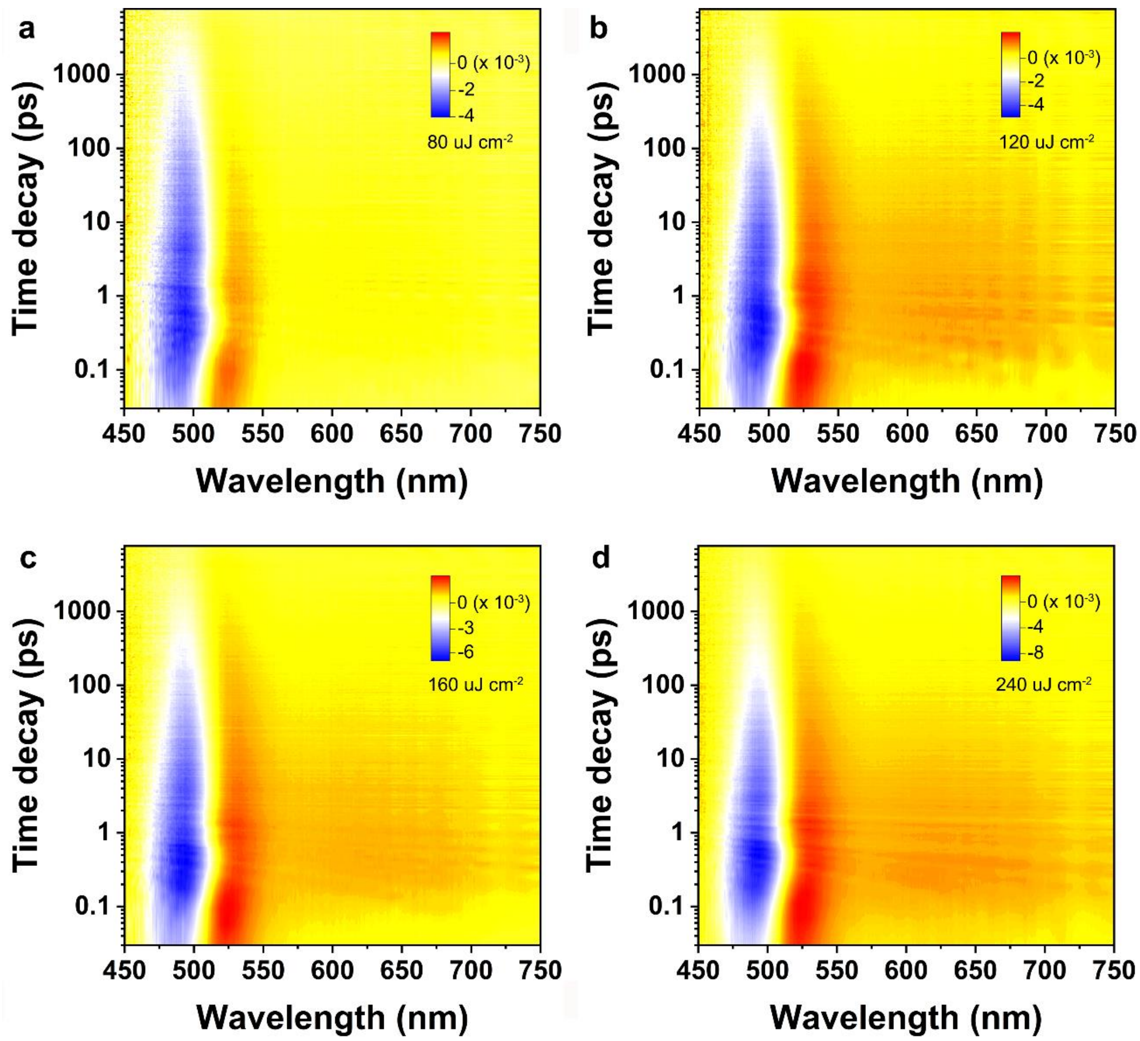

Supplementary Figure 20 Power-dependent femtosecond TA spectra of Au-1 NCs. Femtosecond-TA maps of Au-1 NCs pumped by 400 nm source with the power of (a) 80 μJ cm^{-2}, (b) 120 μJ cm^{-2}, (c) 160 μJ cm^{-2}, and (d) 240 μJ cm^{-2}.

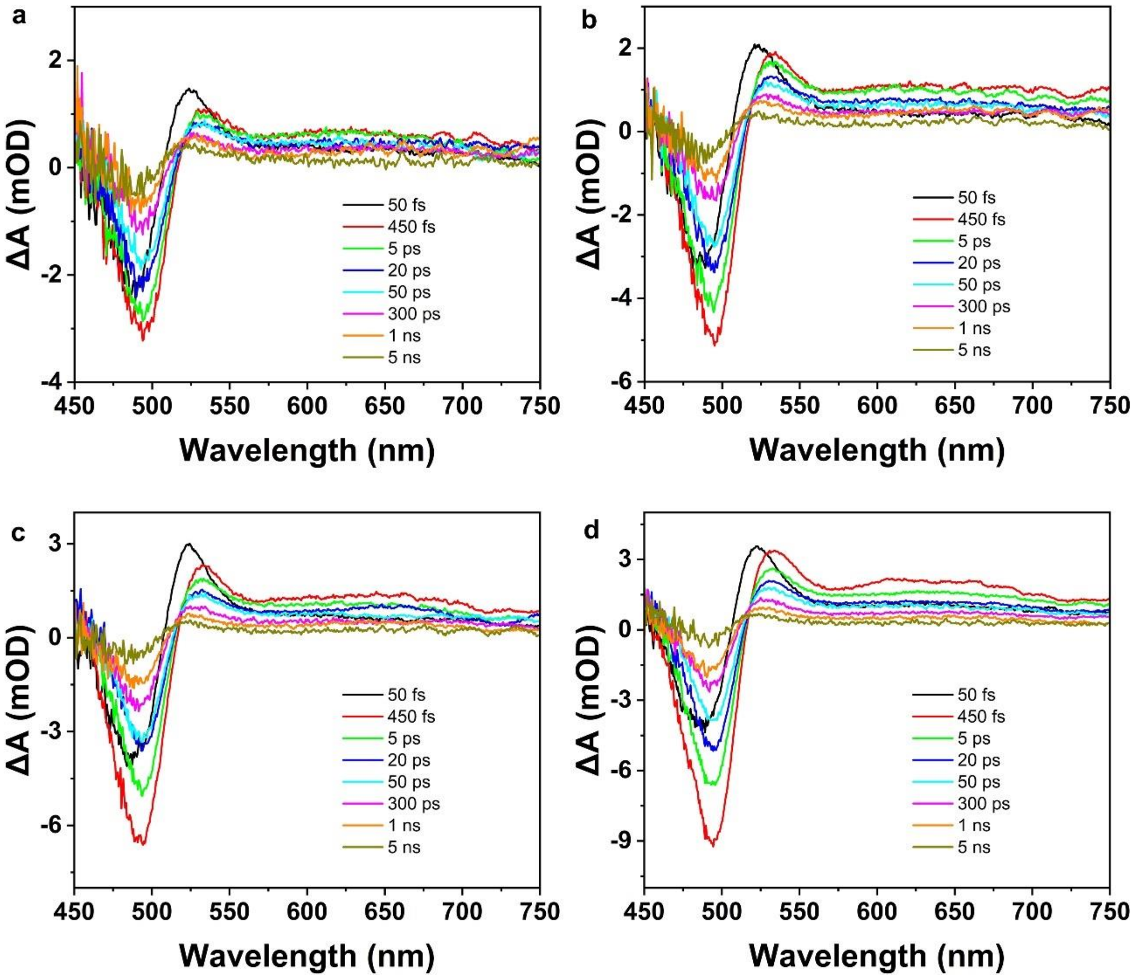

Supplementary Figure 21 Line-by-line TA spectra of Au-1 NCs pumped by different powers. Line-by-line TA spectra within selected time delays from 50 fs to 5 ns of Au-1 NCs pumped by 400 nm source with the power of (a) 80 μJ cm^{-2}, (b) 120 μJ cm^{-2}, (c) 160 μJ cm^{-2}, and (d) 240 μJ cm^{-2}.

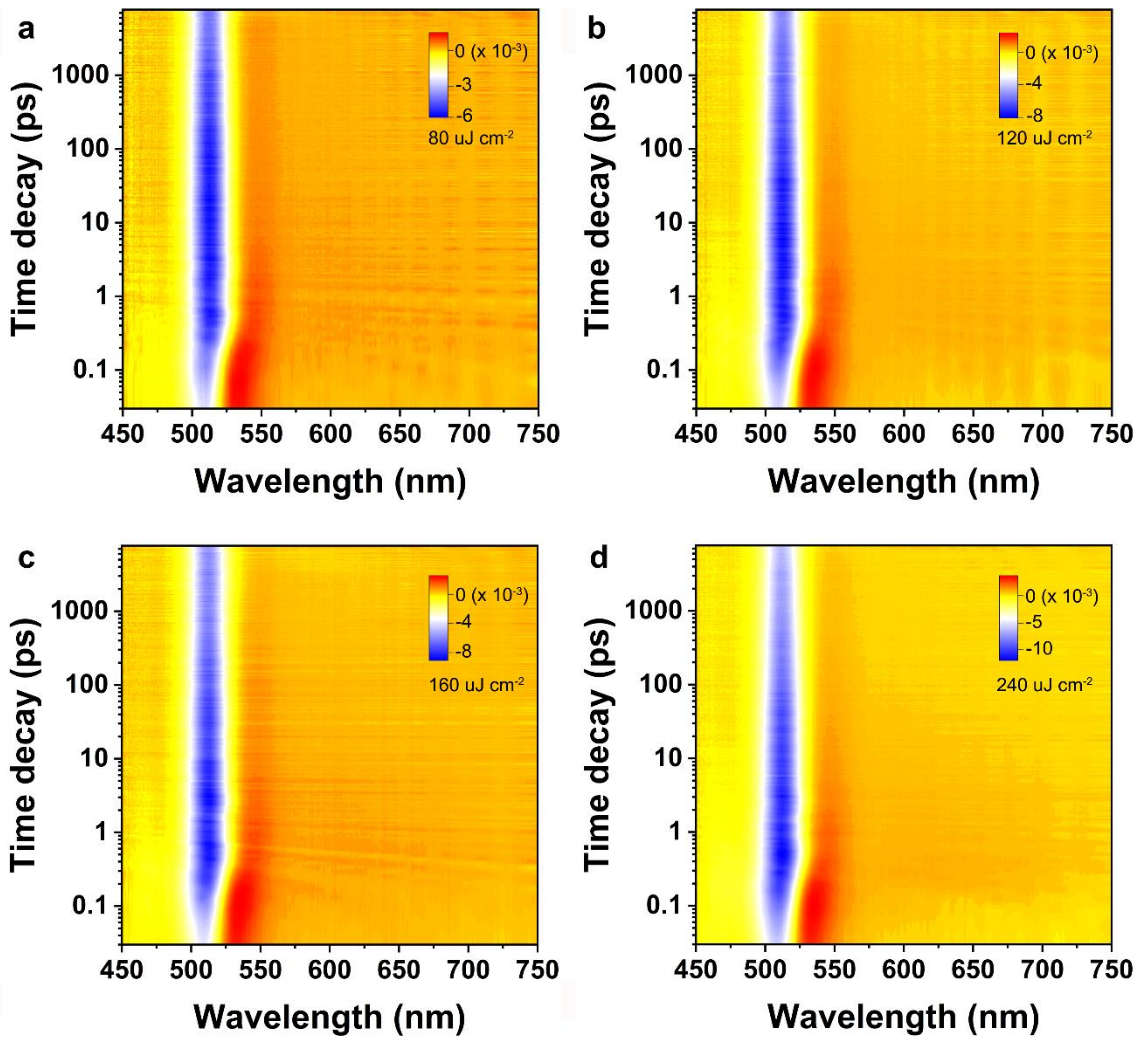

Supplementary Figure 22 Power-dependent femtosecond TA spectra of Au-2 NCs. Femtosecond-TA maps of Au-2 NCs pumped by 400 nm source with the power of (a) 80 μJ cm^{-2}, (b) 120 μJ cm^{-2}, (c) 160 μJ cm^{-2}, and (d) 240 μJ cm^{-2}.

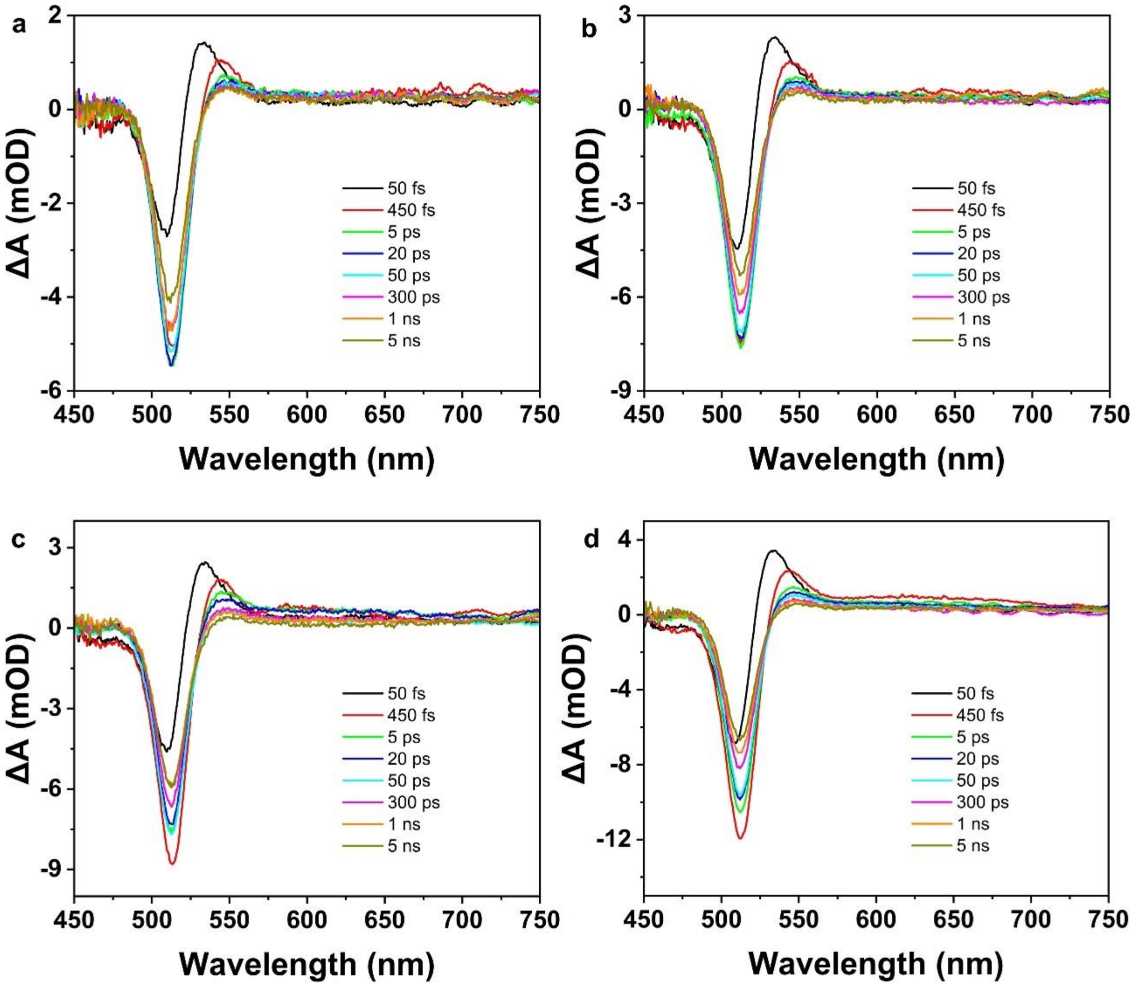

Supplementary Figure 23 Line-by-line TA spectra of Au-2 NCs pumped by different powers. Line-by-line TA spectra within selected time delays from 50 fs to 5 ns of Au-2 NCs pumped by 400 nm source with the power of (a) 80 μJ cm^{-2}, (b) 120 μJ cm^{-2}, (c) 160 μJ cm^{-2}, and (d) 240 μJ cm^{-2}.

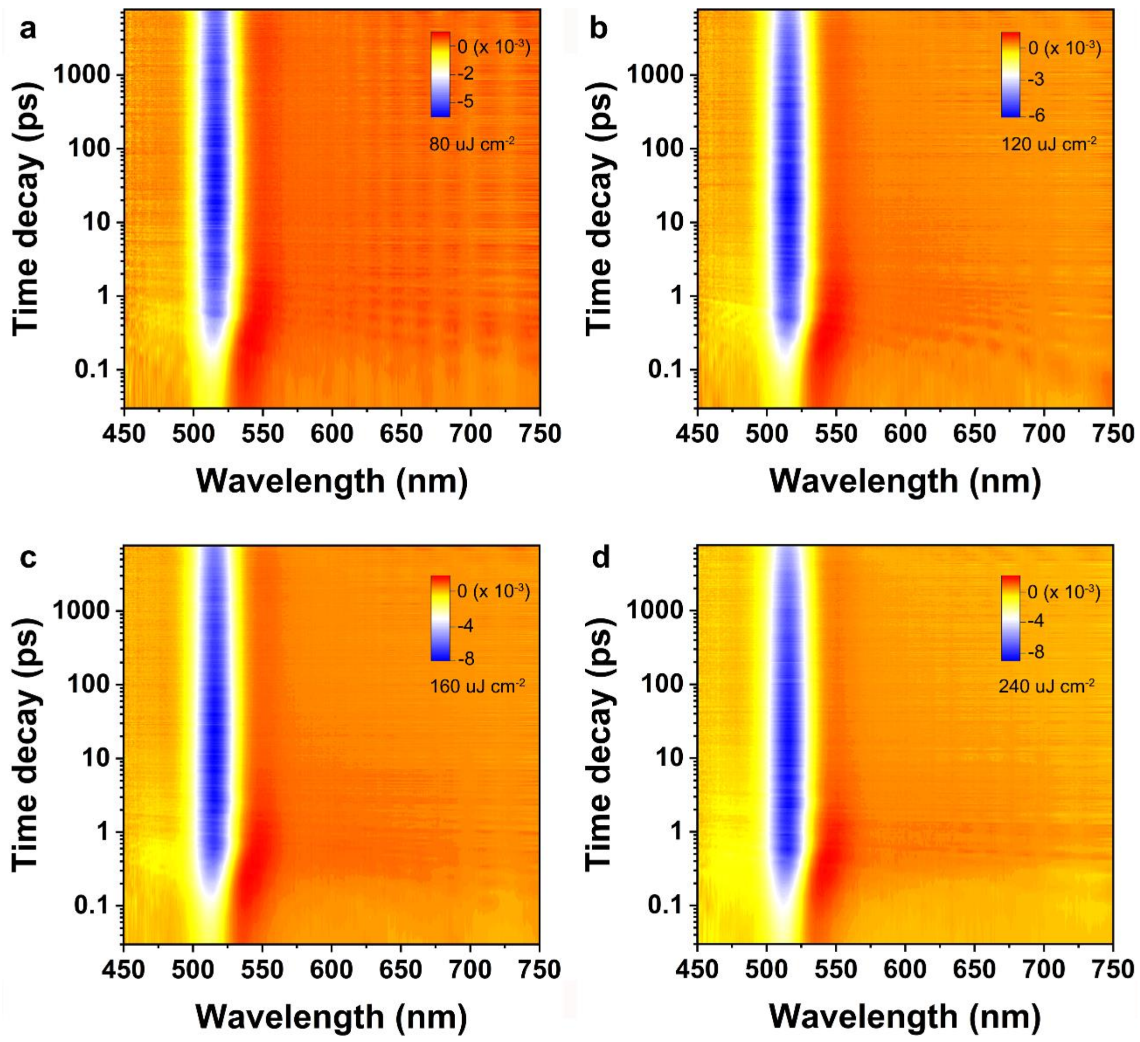

Supplementary Figure 24 Power-dependent femtosecond TA spectra of Au-3 NCs. Femtosecond-TA maps of Au-3 NCs pumped by 400 nm source with the power of (a) 80 μJ cm^{-2}, (b) 120 μJ cm^{-2}, (c) 160 μJ cm^{-2}, and (d) 240 μJ cm^{-2}.

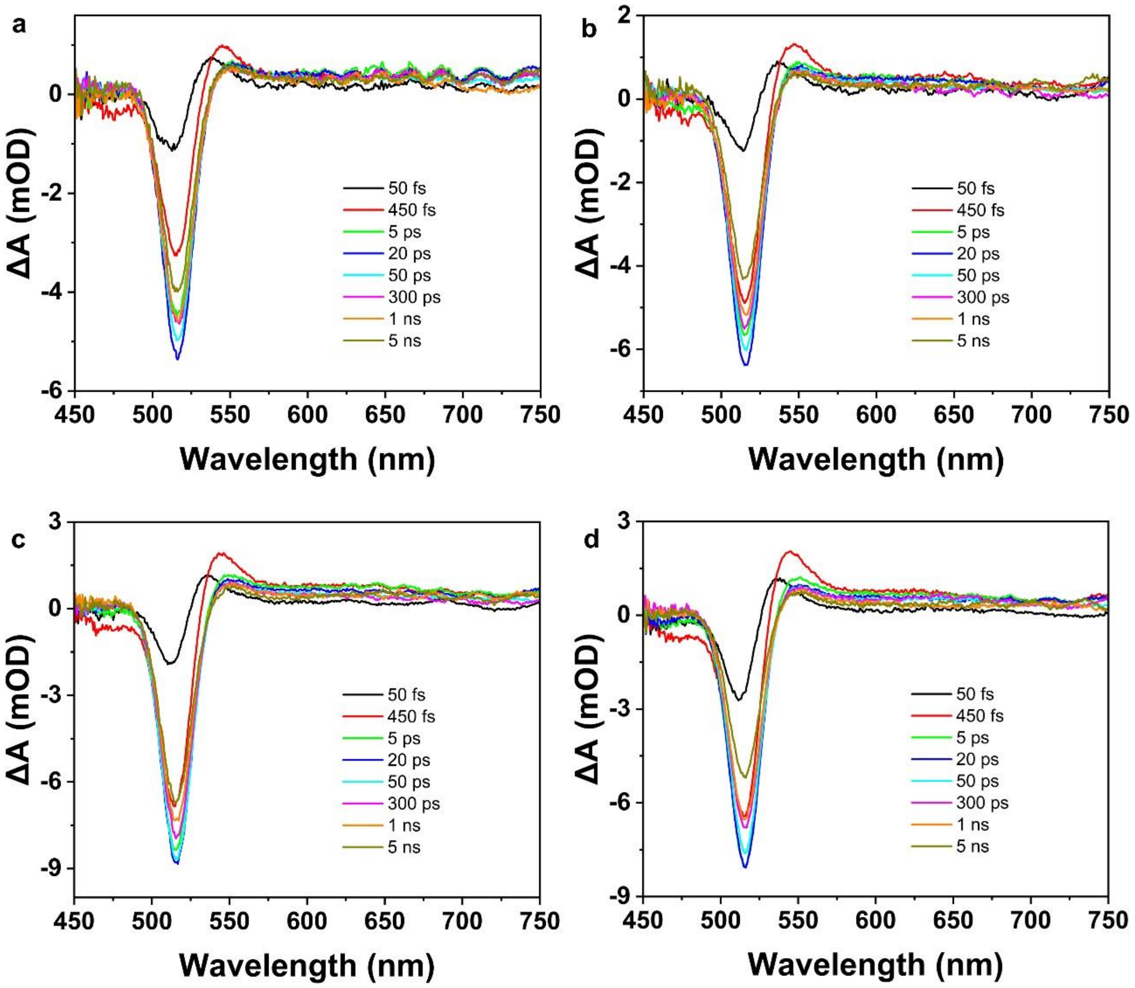

Supplementary Figure 25 Line-by-line TA spectra of Au-3 NCs pumped by different powers. Line-by-line TA spectra within selected time delays from 50 fs to 5 ns of Au-3 NCs pumped by 400 nm source with the power of (a) 80 μJ cm^{-2}, (b) 120 μJ cm^{-2}, (c) 160 μJ cm^{-2}, and (d) 240 μJ cm^{-2}.

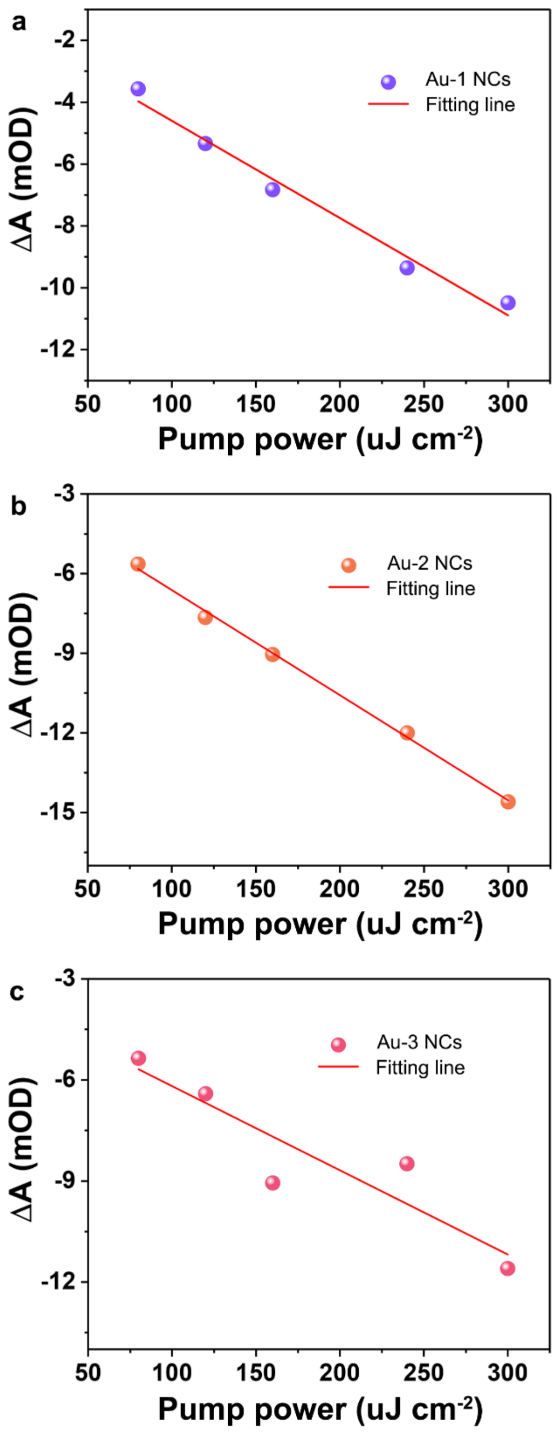

Supplementary Figure 26 The negative-liner relationship of the peak intensity of GSB band as a function of pump power in (a) Au-1, (b) Au-2, and (c) Au-3 NCs. This correlation wipes out the possibility of multiphoton effect and optical damage to the target NCs upon the excitation source of 400 nm.

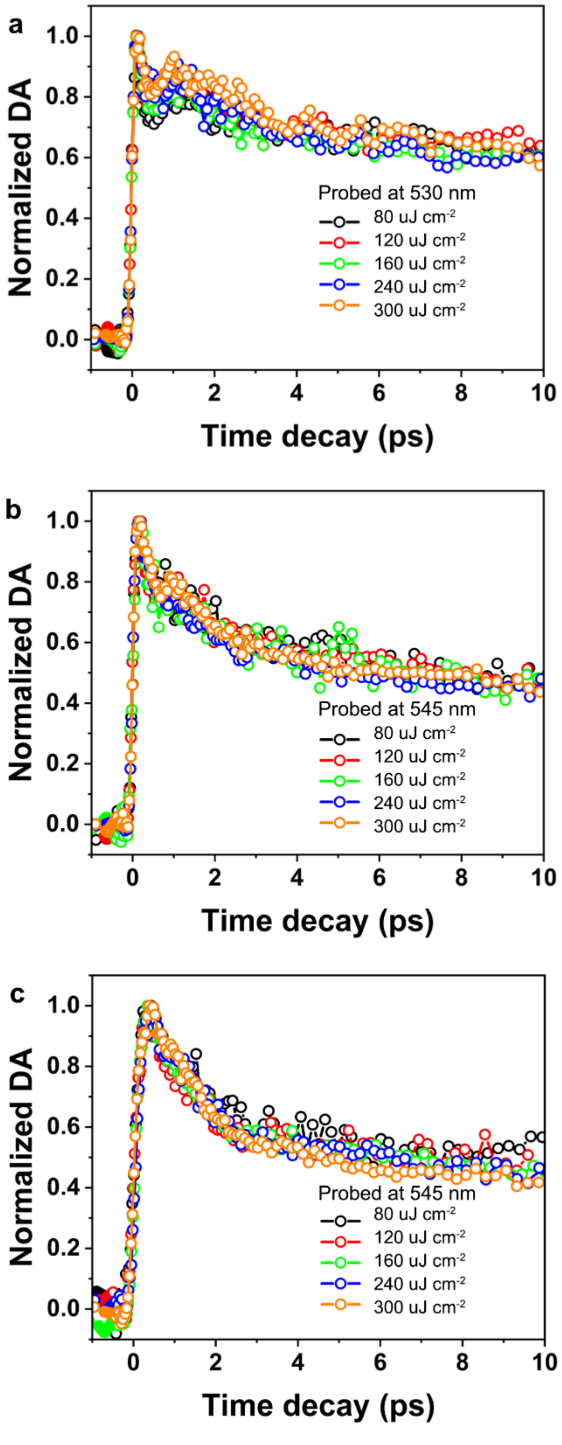

Supplementary Figure 27 Normalized kinetic decays at ESA peak of 530 nm for (a) Au-1 NCs and 545 nm for (b) Au-2 and (c) Au-3 NCs as functions of pump power.

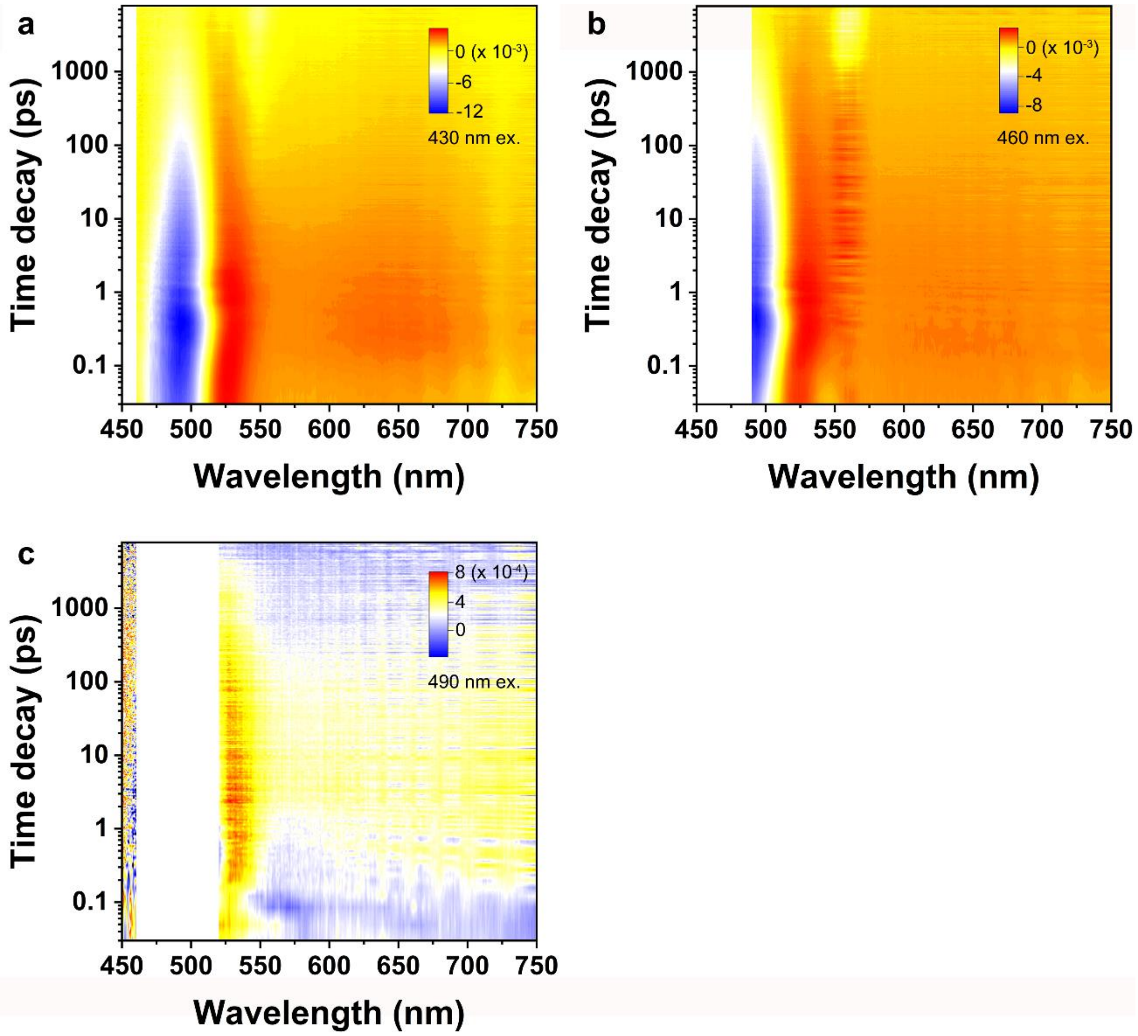

Supplementary Figure 28 Excitation-dependent femtosecond TA spectra of Au-1 NCs. Femtosecond-TA maps of Au-1 NCs pumped by the excitation source of (a) 430 nm, (b) 460 nm, and (c) 490 nm. Scattering signals induced by pump laser were cut off for clarity.

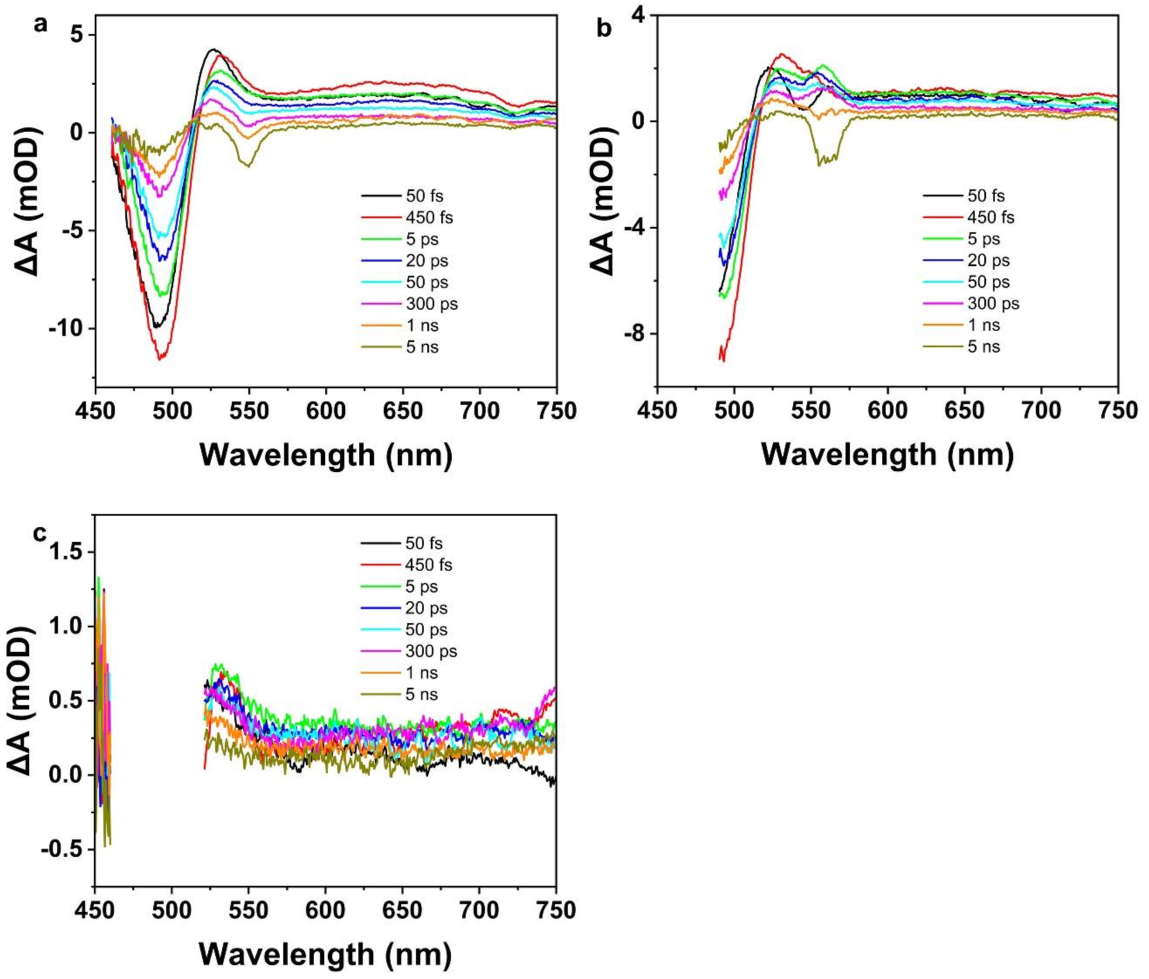

Supplementary Figure 29 Line-by-line TA spectra of Au-1 NCs pumped by different excitation sources. Line-by-line TA spectra within selected time delays from 50 fs to 5 ns of Au-1 NCs pumped by the excitation source of (a) 430 nm, (b) 460 nm, and (c) 490 nm. Scattering signals induced by pump laser were cut off for clarity.

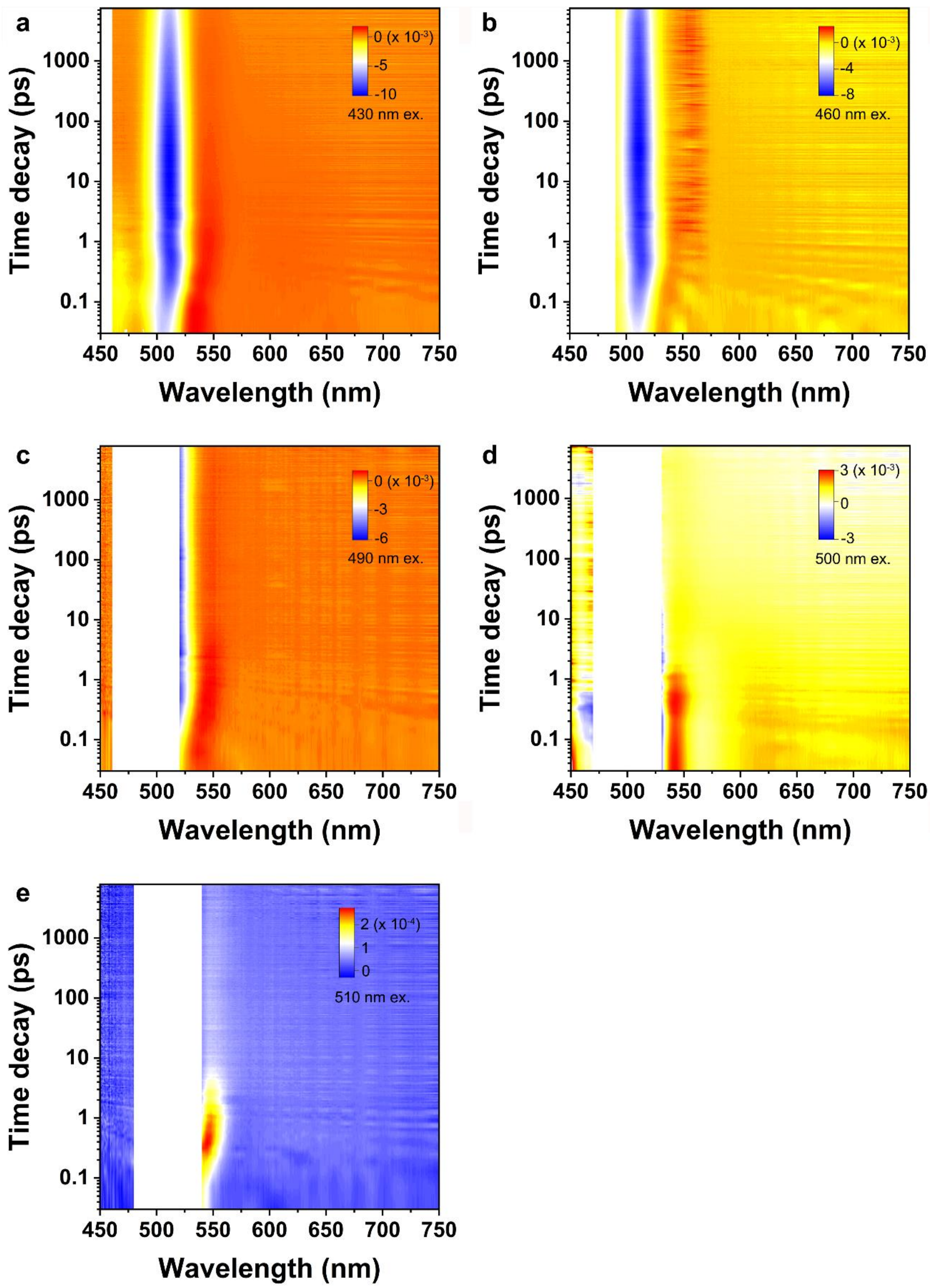

Supplementary Figure 30 Excitation-dependent femtosecond TA spectra of Au-2 NCs. Femtosecond-TA maps of Au-2 NCs pumped by the excitation source of (a) 430 nm, (b) 460 nm, (c) 490 nm, (d) 500 nm, and (e) 510 nm. Scattering signals induced by the pump laser were cut off for clarity.

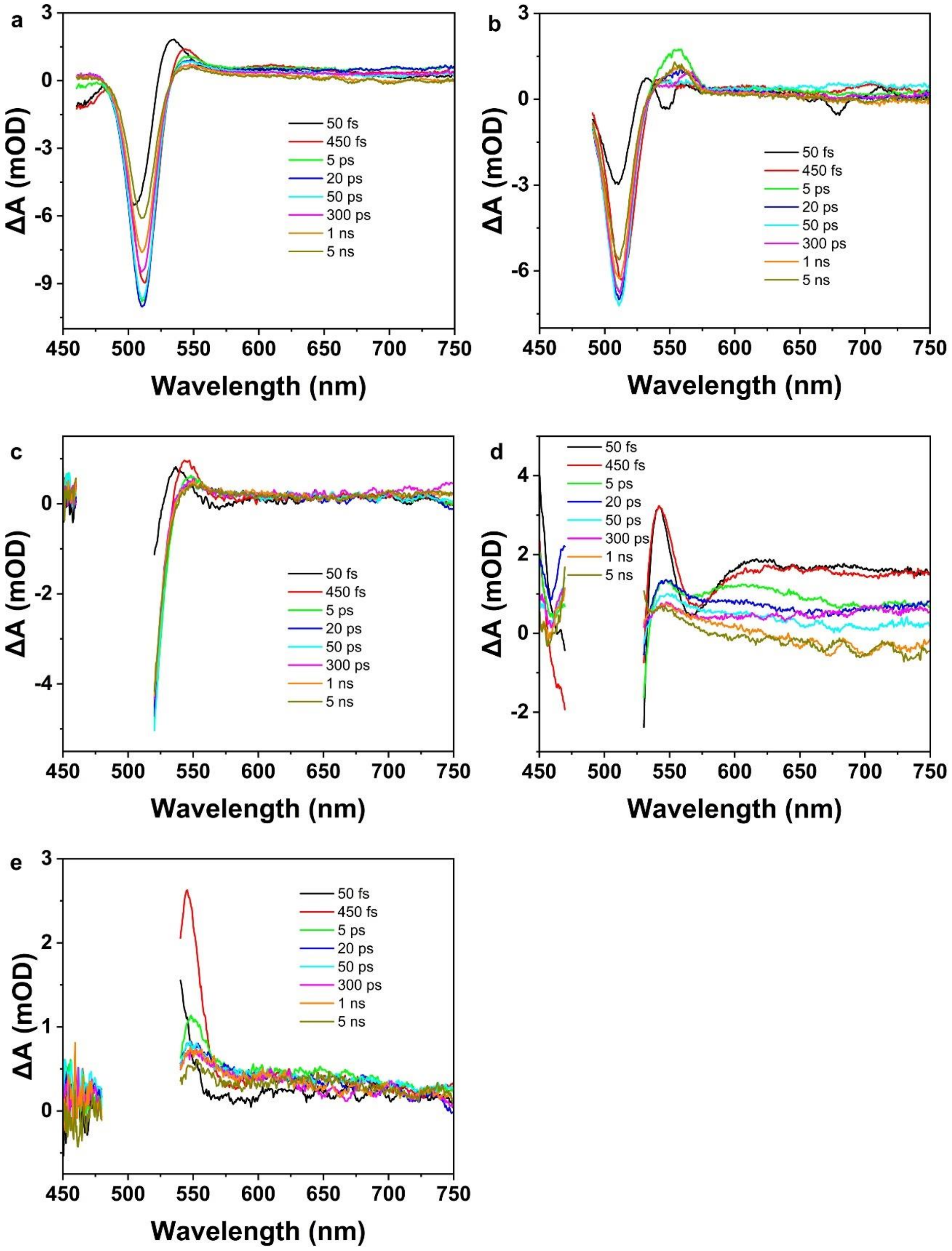

Supplementary Figure 31 Line-by-line TA spectra of Au-2 NCs pumped by different excitation sources. Line-by-line TA spectra within selected time delays from 50 fs to 5 ns of Au-2 NCs pumped by the excitation source of (a) 430 nm, (b) 460 nm, (c) 490 nm, (d) 500 nm, and (e) 510 nm. Scattering signals induced by pump laser were cut off for clarity.

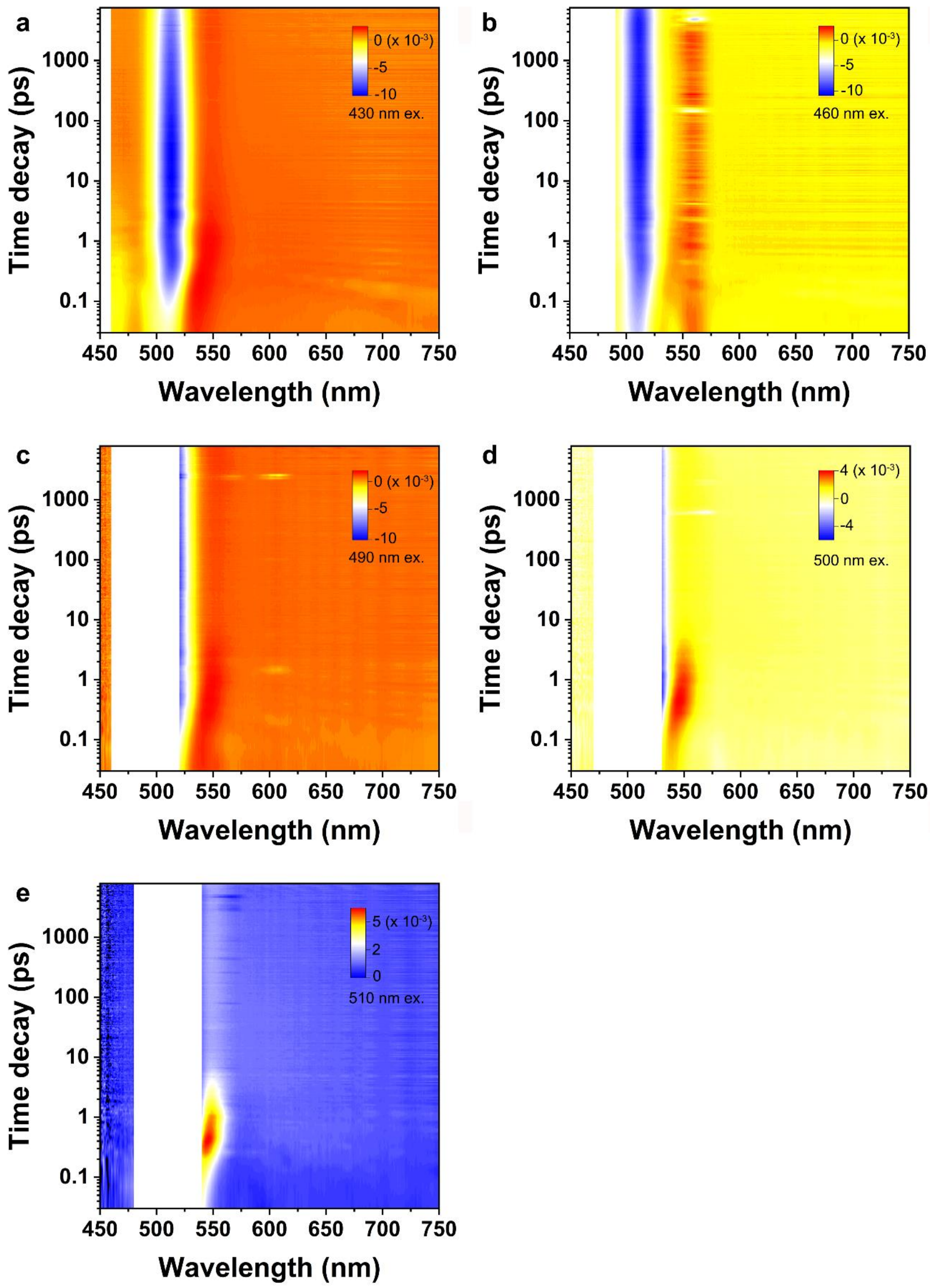

Supplementary Figure 32 Excitation-dependent femtosecond TA spectra of Au-3 NCs. Femtosecond-TA maps of Au-3 NCs pumped by the excitation source of (a) 430 nm, (b) 460 nm, (c) 490 nm, (d) 500 nm, and (e) 510 nm. Scattering signals induced by pump laser were cut off for clarity.

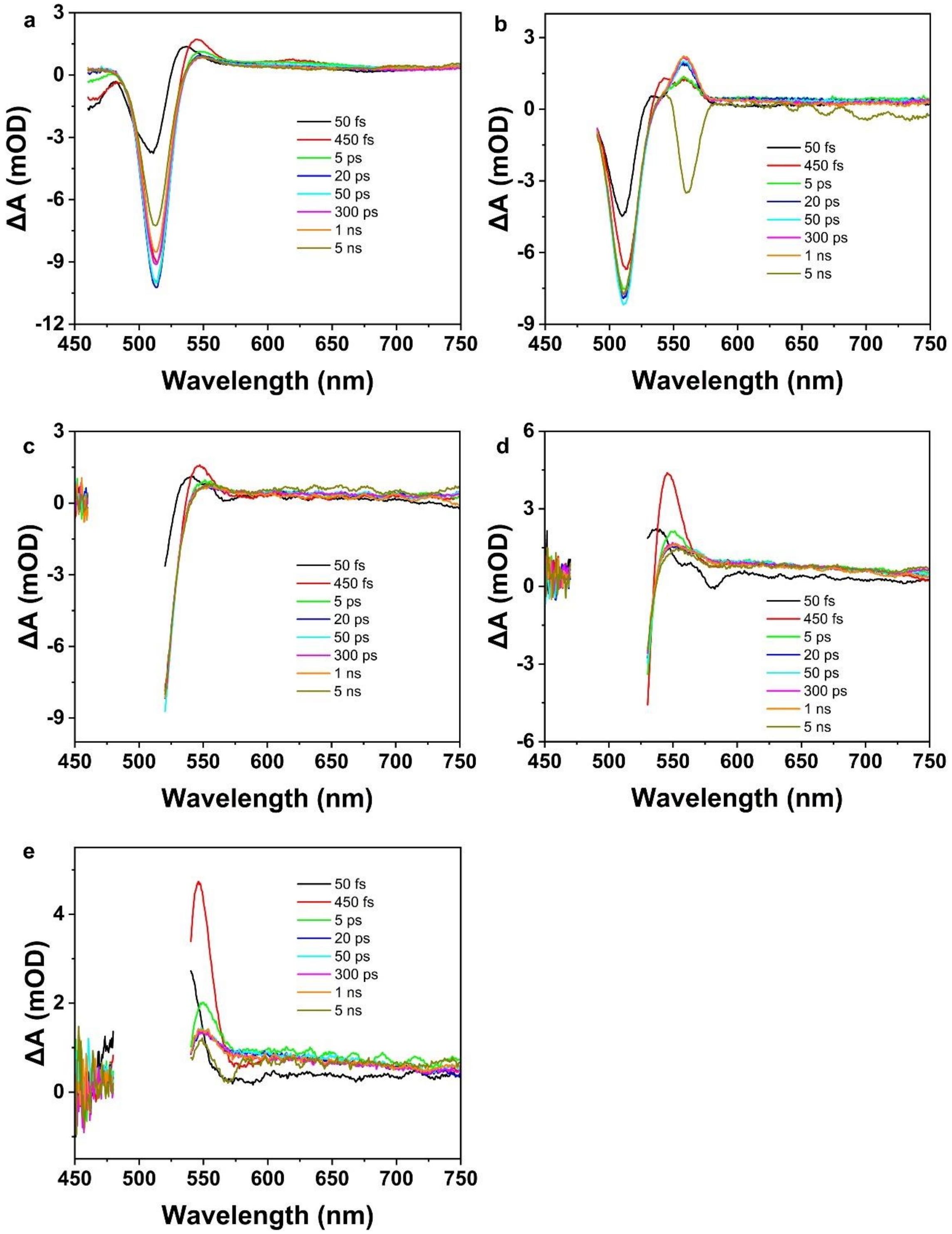

Supplementary Figure 33 Line-by-line TA spectra of Au-3 NCs pumped by different excitation sources. Line-by-line TA spectra within selected time delays from 50 fs to 5 ns of Au-3 NCs pumped by the excitation source of (a) 430 nm, (b) 460 nm, (c) 490 nm, (d) 500 nm, and (e) 510 nm. Scattering signals induced by pump laser were cut off for clarity.

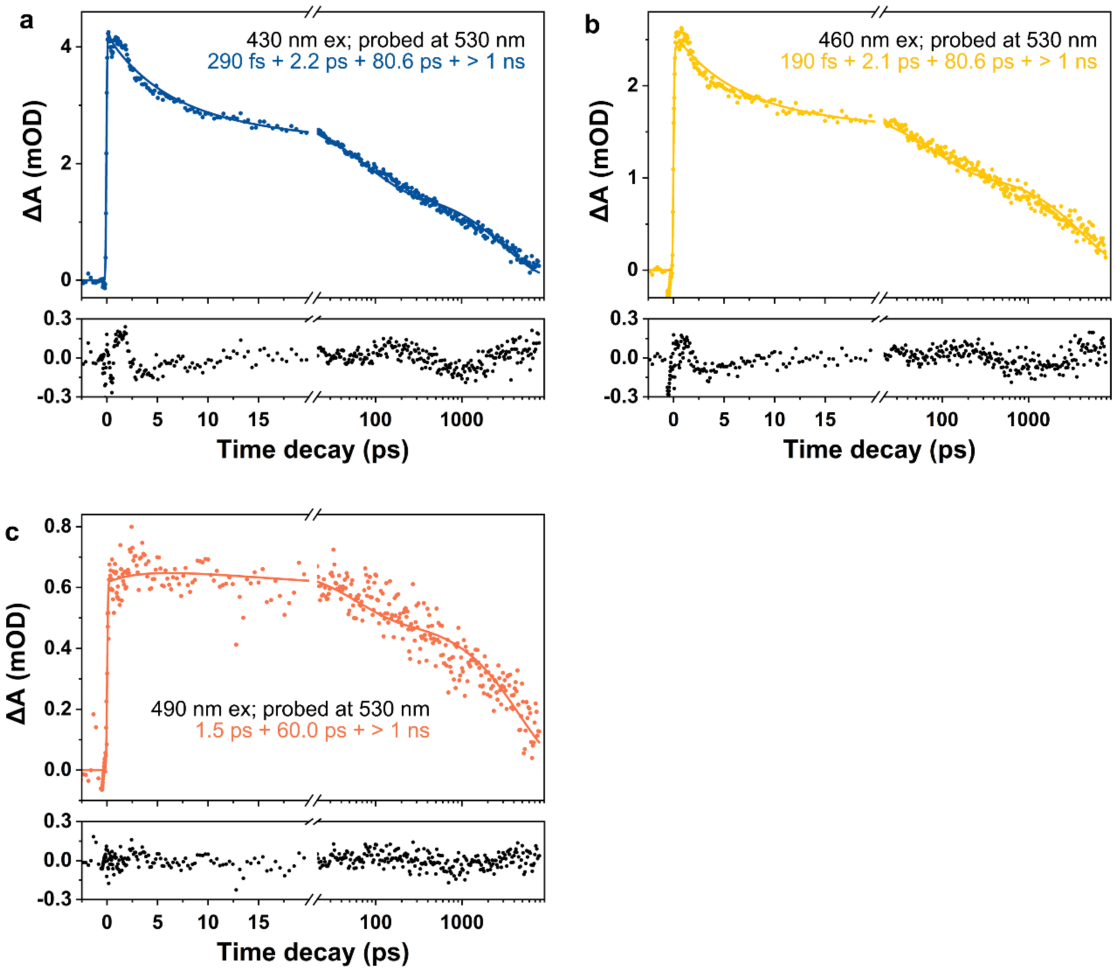

Supplementary Figure 34 Kinetic decays of Au-1 NCs pumped by different excitation sources. Kinetic decays around 530 nm and corresponding fitting lines and residuals extracted from the global fitting of TA data map of Au-1 NCs pumped by (a) 430 nm, (b) 460 nm, and (c) 490 nm.

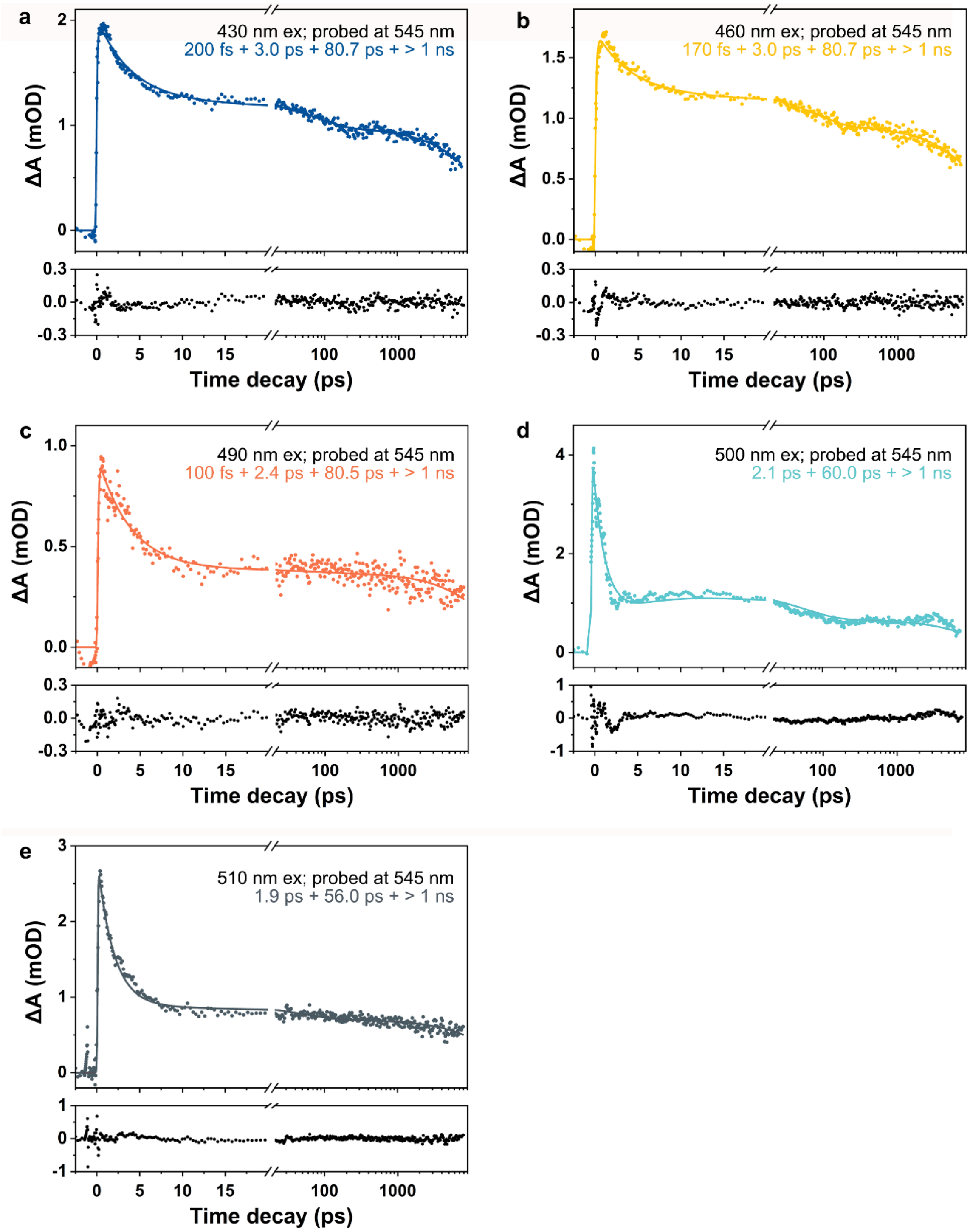

Supplementary Figure 35 Kinetic decays of Au-2 NCs pumped by different excitation sources. Kinetic decays around 545 nm and corresponding fitting lines and residuals extracted from the global fitting of TA data map of Au-2 NCs pumped by (a) 430 nm, (b) 460 nm, (c) 490 nm, (d) 500 nm, and (e) 510 nm.

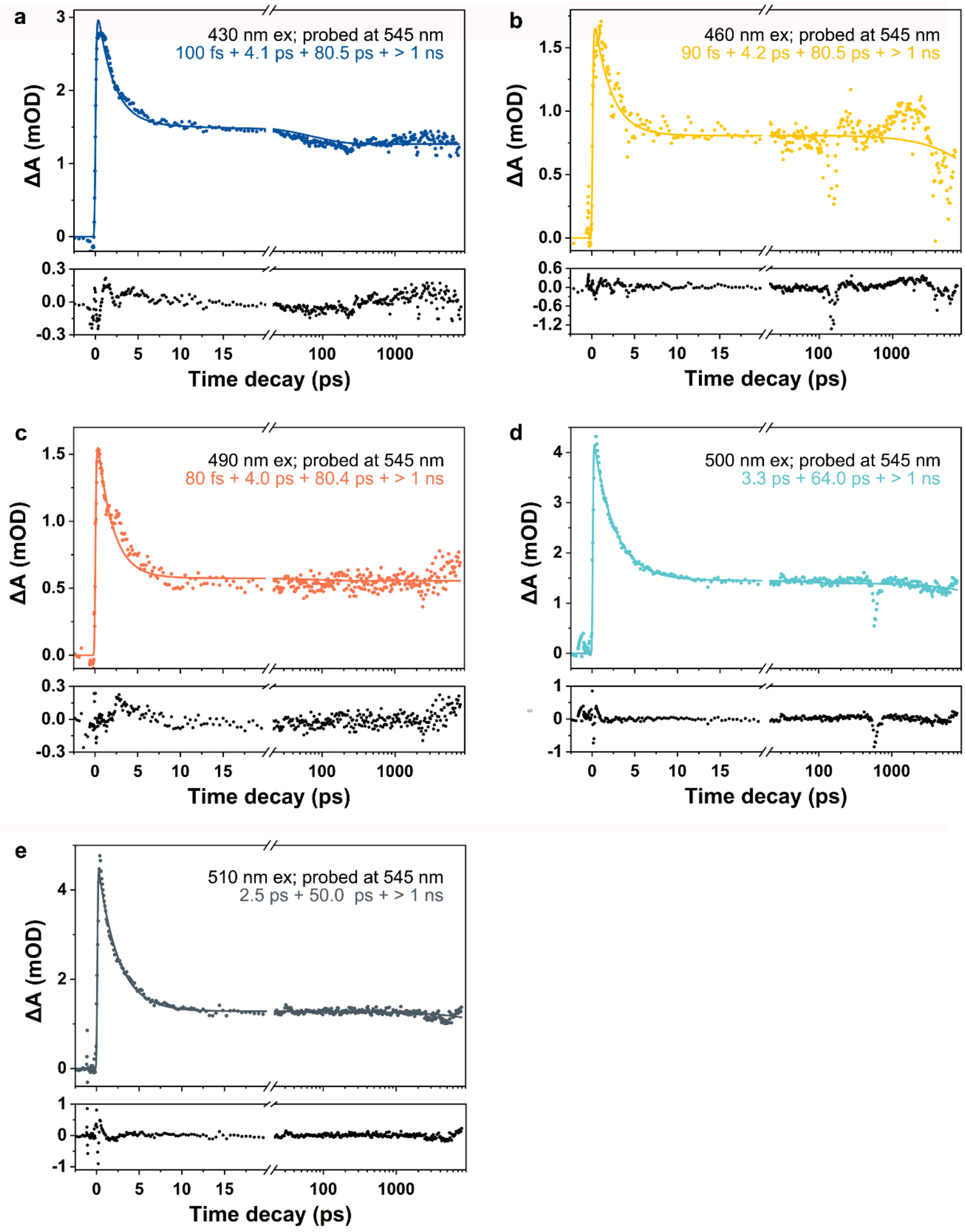

Supplementary Figure 36 Kinetic decays of Au-3 NCs pumped by different excitation sources. Kinetic decays around 545 nm and corresponding fitting lines and residuals extracted from the global fitting of TA data map of Au-3 NCs pumped by (a) 430 nm, (b) 460 nm, (c) 490 nm, (d) 500 nm, and (e) 510 nm.

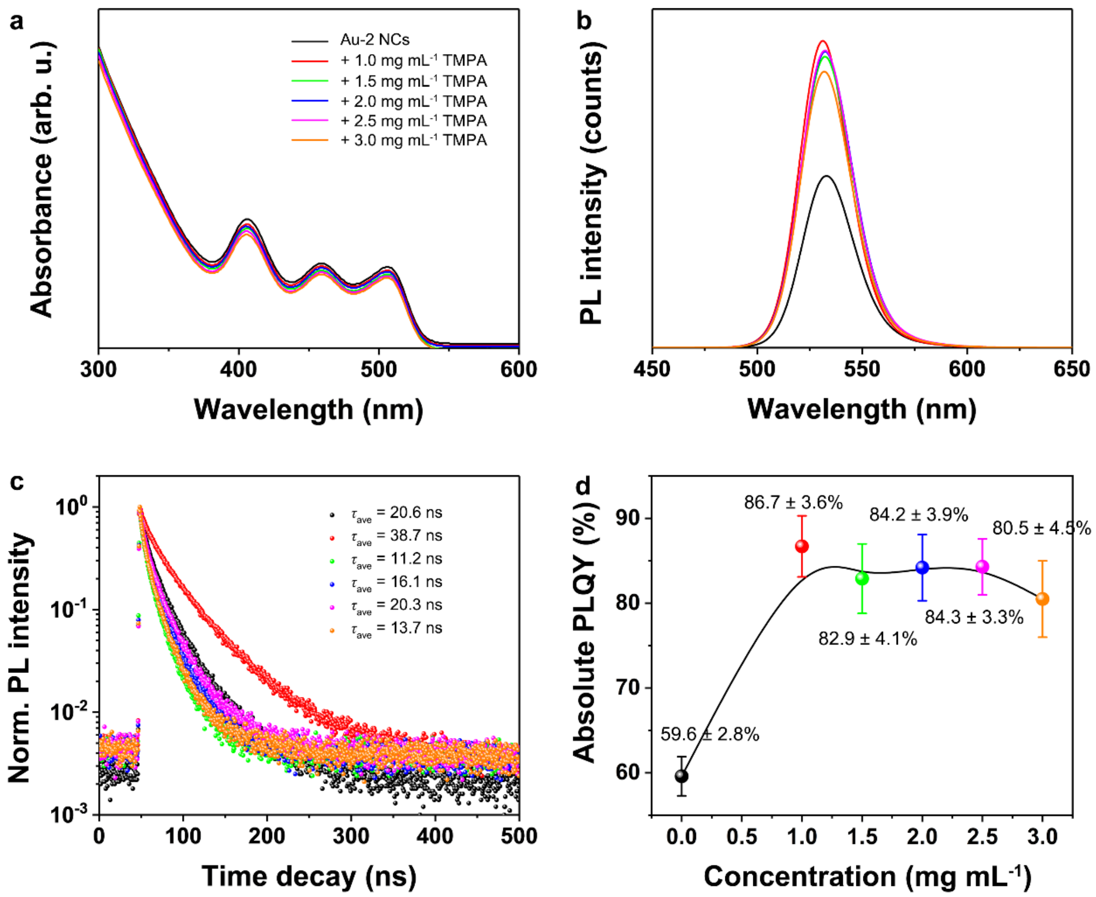

Supplementary Figure 37 Optical properties of Au-2 NCs added with different amount of TMPA. (a) UV-vis spectra, (b) PL spectra, and (c) PL time-resolved spectra of as-synthesized Au-2 NCs added with different concentrations of TMPA ligand. (d) The recorded absolute PLQY in Au-2-TMPA NCs as a function of the concentration of TMPA ligand. As for the PL measurements, the ODs at 405 nm of all samples were fixed at 0.1 by varying the concentration of NCs in water. The absolute PLQY was measured at the same OD value of 0.1. For the additional discussion, Au-2-TMPA NCs exclusively refer to Au-2 NCs added with 1.0 mg mL^{-1} TMPA ligand because it possesses the best PL performance.

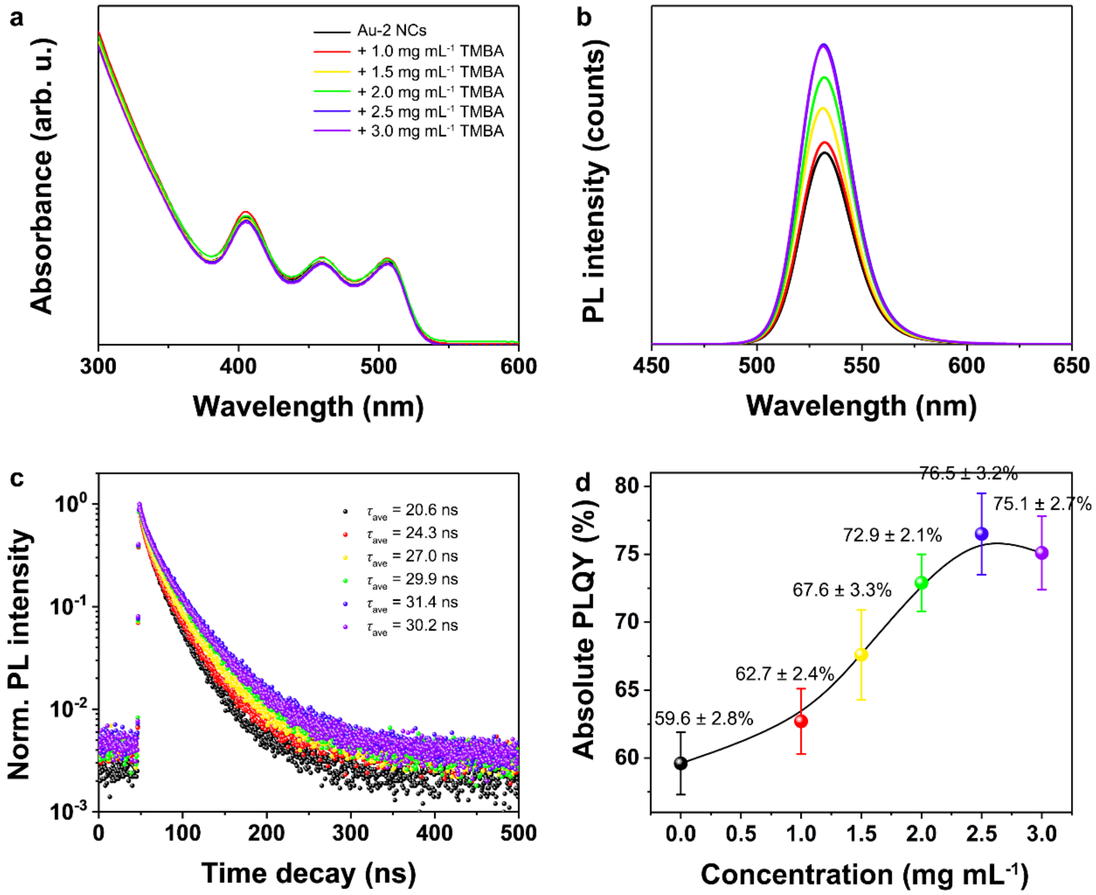

Supplementary Figure 38 Optical properties of Au-2 NCs added with different amount of TMBA. (a) UV-vis spectra, (b) PL spectra, and (c) PL time-resolved spectra of as-synthesized Au-2 NCs added with different concentrations of TMBA ligand. (d) The recorded absolute PLQY in Au-2-TMBA NCs as a function of the concentration of TMBA ligand. For the additional discussion, Au-2-TMBA NCs exclusively refer to Au-2 NCs added with 2.5 mg mL^{-1} TMBA ligand because it possesses the best PL performance.

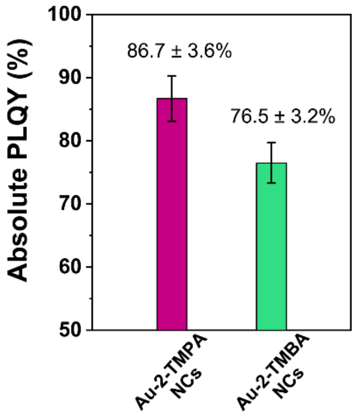

Supplementary Figure 39 The statistics of recorded PLQY values of 86.7 ± 3.6% and 76.5 ± 3.2% for Au-2-TMPA NCs and Au-2-TMBA NCs, respectively. The absolute PLQY values were directly measured on a FLS1000 spectrofluorometer attached with an integrating sphere.

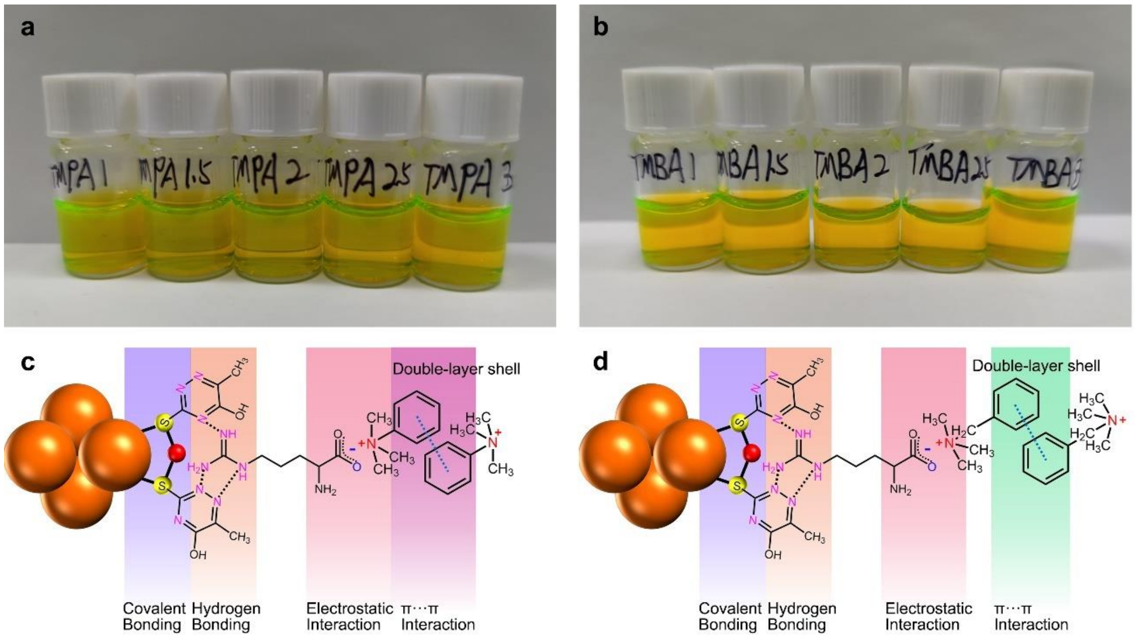

Supplementary Figure 40 Proposed surface structure of other two gold NCs. Digital optical photographs of the aqueous solutions of Au-2 NCs anchored with different concentrations (from left to right: 1.0, 1.5, 2.0, 2.5, and 3.0 mg mL^{-1}) of (a) TMPA and (b) TMBA ligands. (c) and (d) are the corresponding schematic illustration of the double-layer shell structure of Au-2-TMPA and Au-2-TMBA NCs in an aqueous solution.

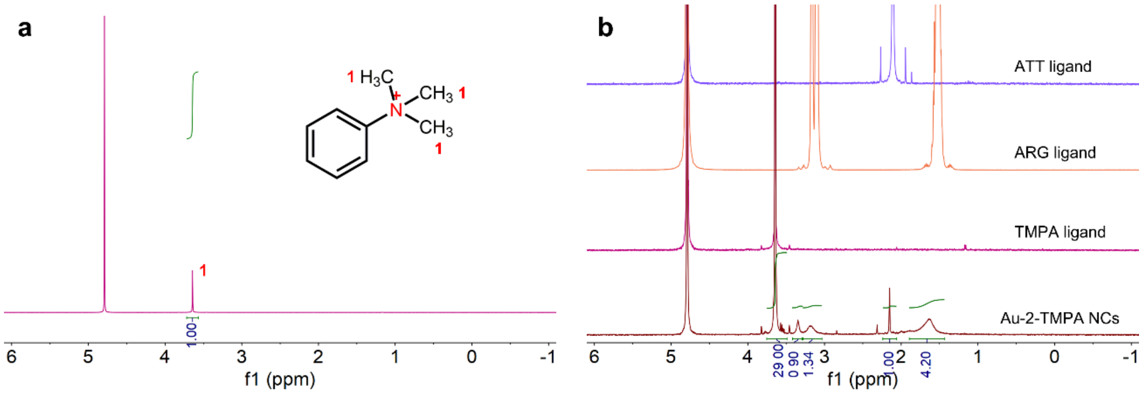

Supplementary Figure 41 ^{1}H-NMR spectra of (a) TMPA ligand, and (b) the comparison of ^{1}H-NMR spectra between peripheral ATT, ARG, TMPA ligands, and Au-2-TMPA NCs.

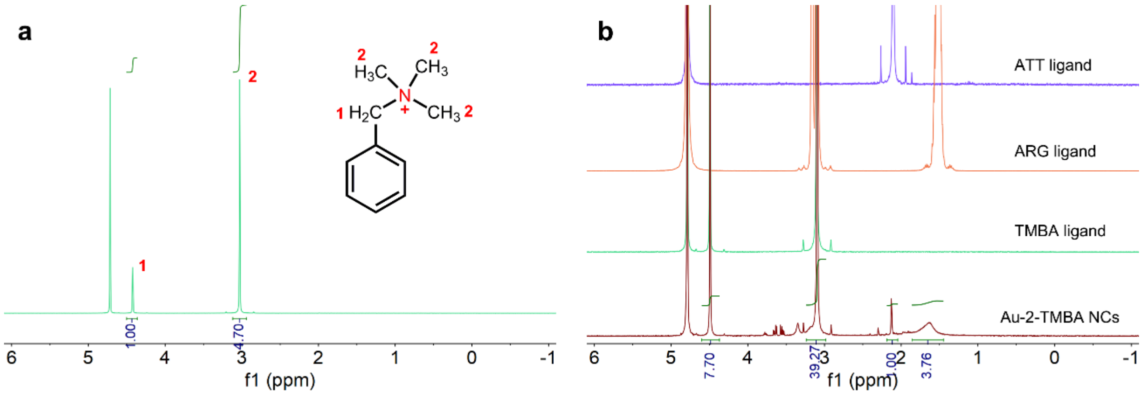

Supplementary Figure 42 ^{1}H-NMR spectra of (a) TMBA ligand, and (b) the comparison of ^{1}H-NMR spectra between peripheral ATT, ARG, TMBA ligands, and Au-2-TMBA NCs.

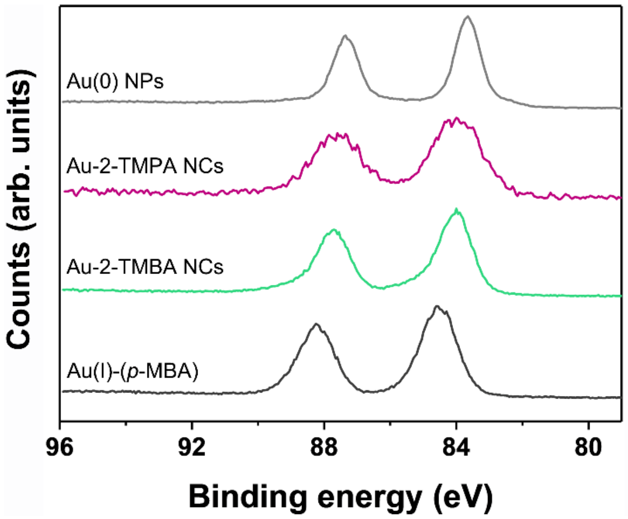

Supplementary Figure 43 Au 4*f* XPS spectra of Au(0) NPs, Au-2-TMPA NCs, Au-2-TMBA NCs, and Au(I)-(*p*-MBA) complexes.

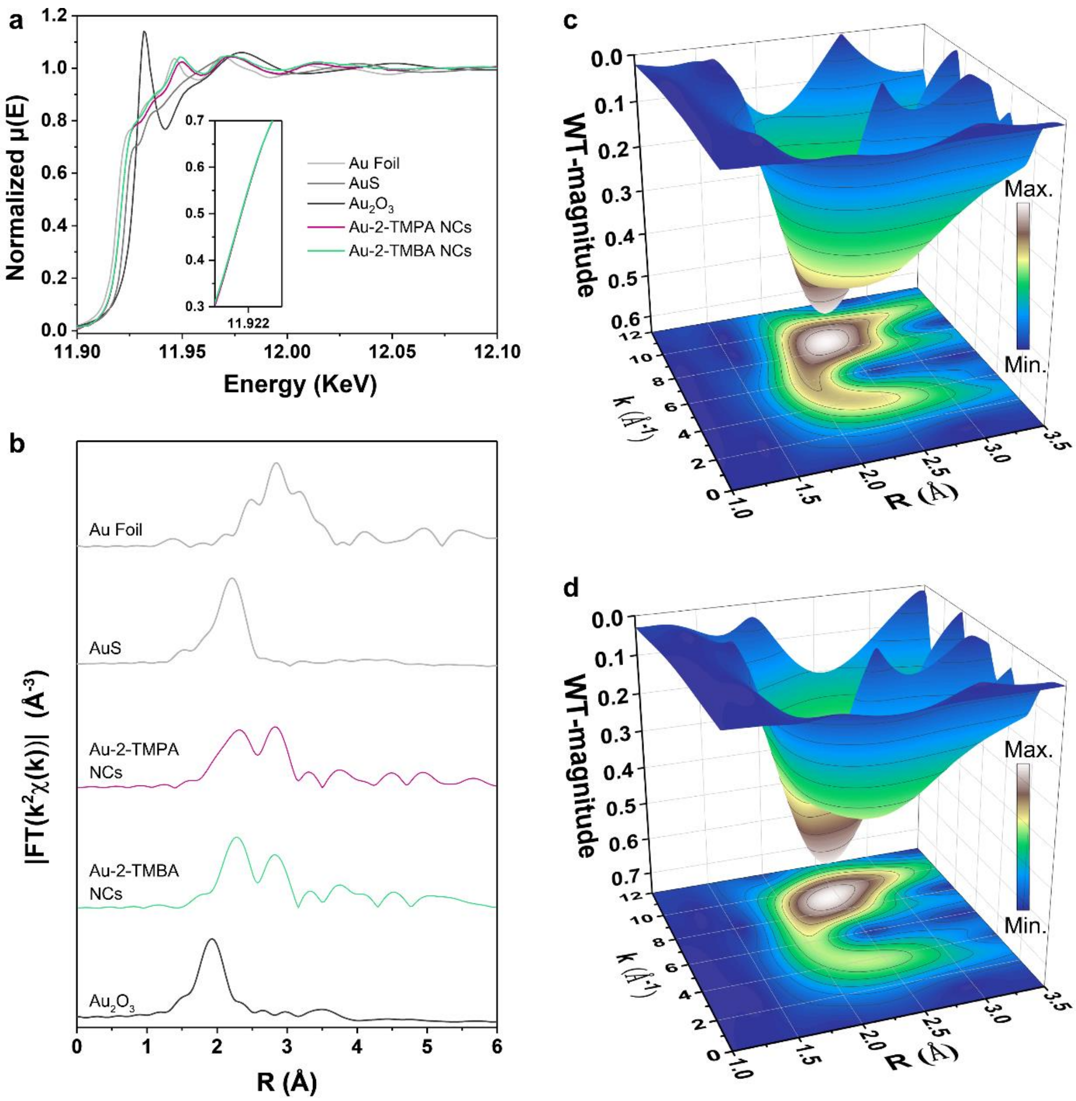

Supplementary Figure 44 Local structures of other two gold NCs. (a) Au L_3-edge XANES and (b) Au L_3-edge FT-EXAFS spectra of Au foil, AuS, Au_2O_3, Au-2-TMPA, and Au-2-TMPA NCs. (c) and (d) are the 3D WT-extended EXAFS spectra of Au-2-TMPA and Au-2-TMPA NCs. Two scattering path signals are detected at [4.2, 2.42], [8.8, 2.76], and [4.2, 2.42], [9.0, 2.76] in Au-2-TMPA and Au-2-TMPA NCs, respectively, which are assigned to be Au-S and Au-Au bonding, respectively.

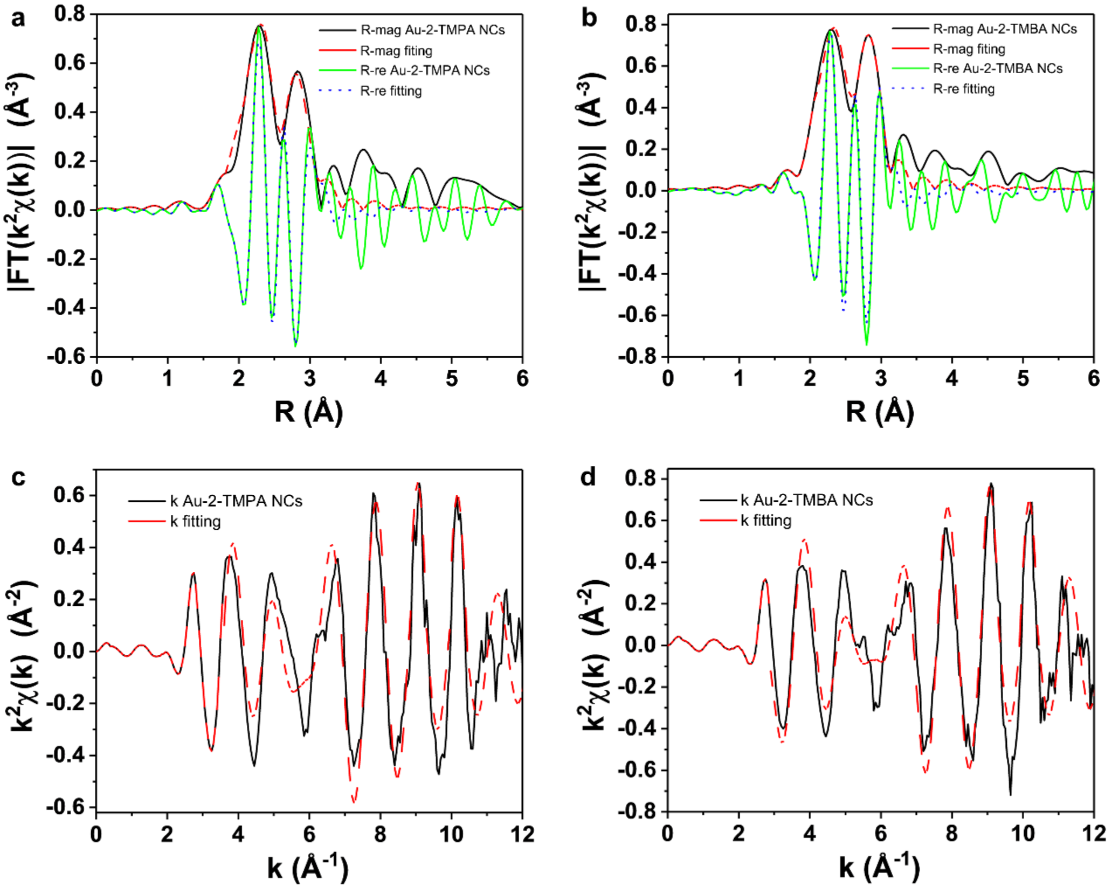

Supplementary Figure 45 FT of the k^2-weighted EXAFS spectrra and their corresponding fitting in R space of (a) Au-2-TMPA and (b) Au-2-TMBA NCs with the magnitude and real component. (c) and (d) are $k^2\chi(k)$ space spectra and their corresponding fitting of Au-2-TMPA and Au-2-TMBA NCs.

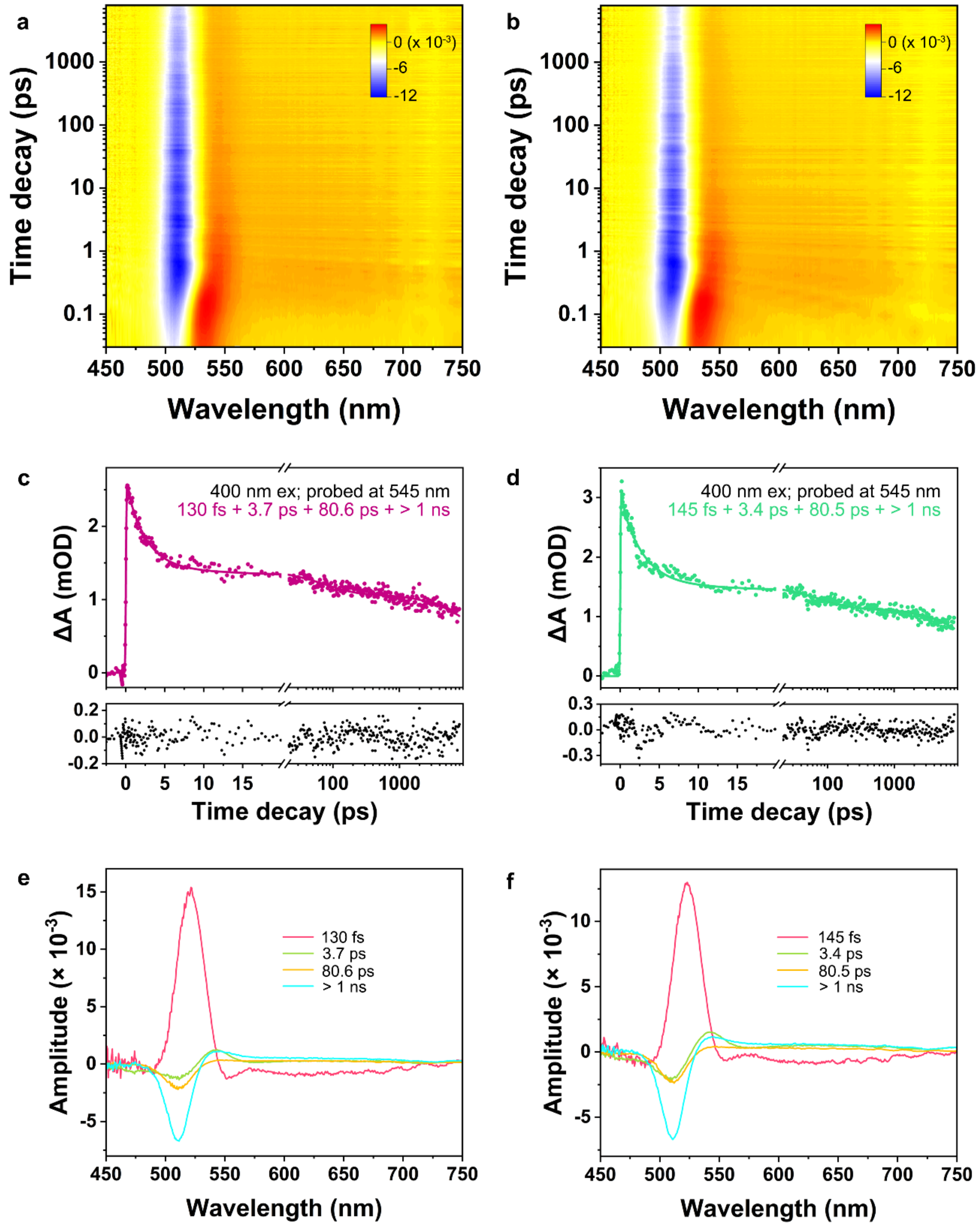

Supplementary Figure 46 Ultrafast electron dynamics of other two gold NCs. Femtosecond-TA maps of (a) Au-2-TMPA and (b) Au-2-TMBA NCs pumped by 400 nm light source. Selected kinetic decays of (c) Au-2-TMPA and (d) Au-2-TMBA NCs probed around 545 nm, and their corresponding DAS spectra (e, f) were obtained from global fittings on the femtosecond-TA map data. The fitted-out amplitudes of IC and structural relaxation components are smaller than pristine Au-2 NCs, suggesting the suppressed non-radiative relaxations.

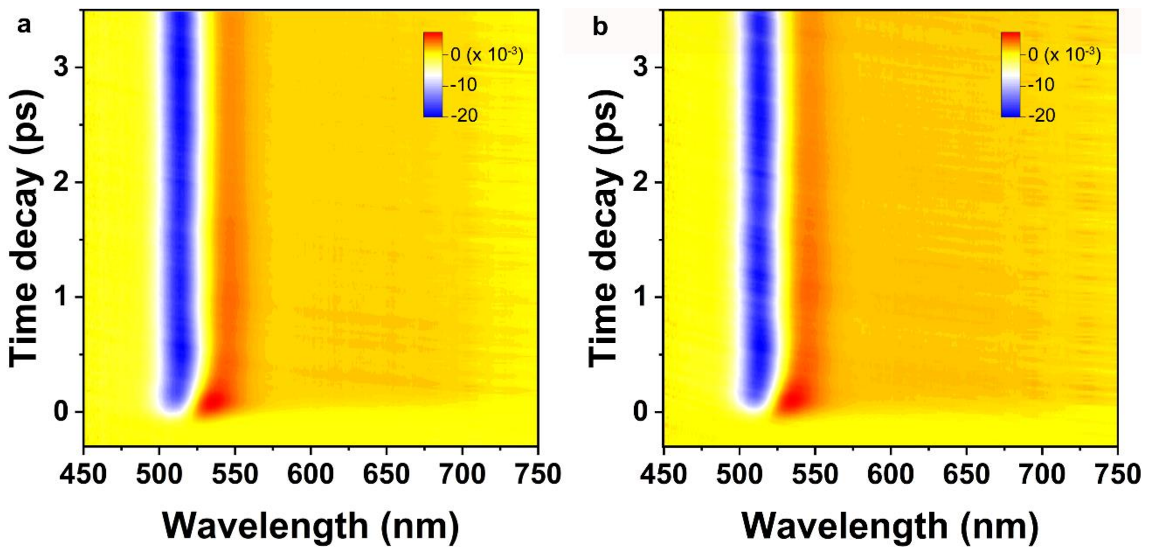

Supplementary Figure 47 Short-range TA data maps of (a) Au-2-TMPA and (b) Au-2-TMBA NCs between -0.5 ps and 4.3 ps upon the pumping laser of 400 nm.

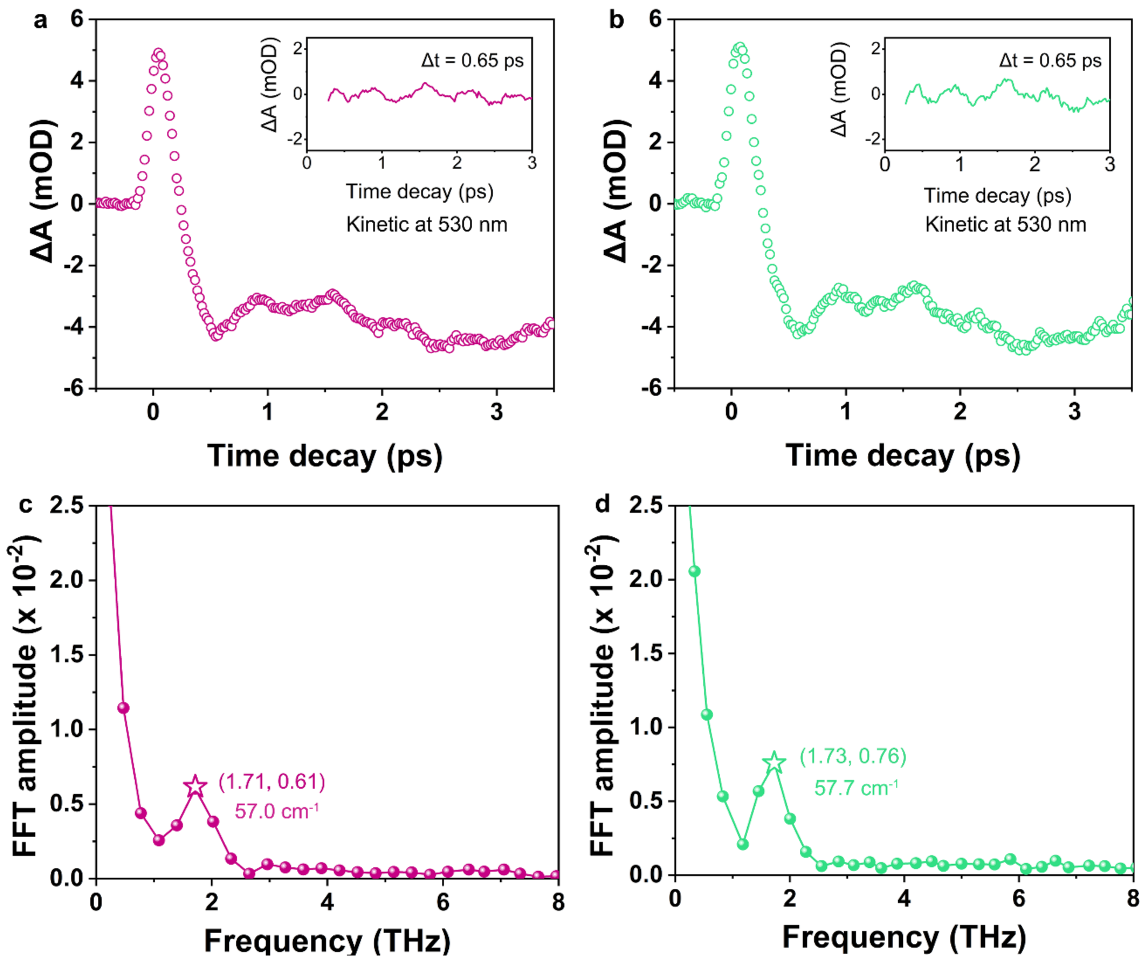

Supplementary Figure 48 Kinetic traces at zero positions (upper panel) in TA maps and corresponding FFT results by plotting the amplitude as a function of frequency (bottom panel) of (a, c) Au-2-TMPA and (b, d) Au-2-TMBA NCs.

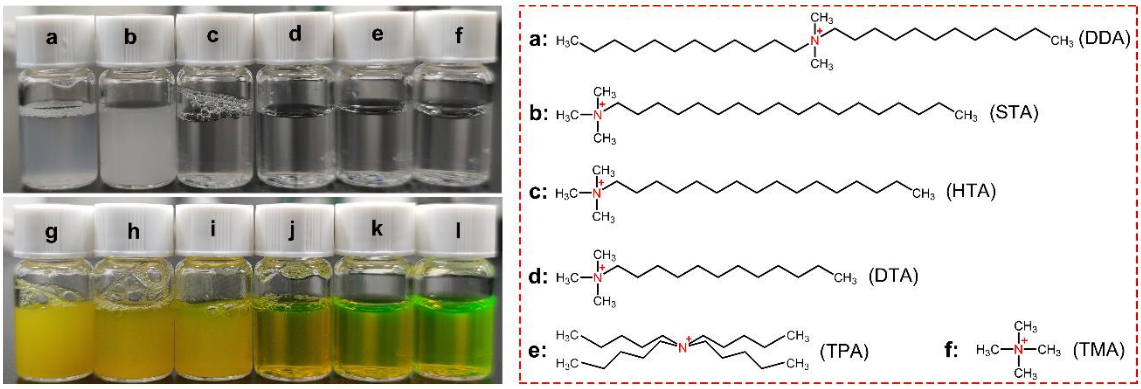

Supplementary Figure 49 Digital optical photographs of the aqueous solutions of (a) DDA bromide, (b) STA bromide, (c) HTA bromide, (d) DTA chloride, (e) TPA bromide and (f) TMA chloride (upper left). The products of (g) DDA bromide, (h) STA bromide, (i) HTA bromide, (j) DTA chloride, (k) TPA bromide, and (l) TMA chloride reacted with Au-2 NCs for 30 min (bottom left). The corresponding structural formulas of as-used DDA, STA, HTA, DTA, TPA, and TMA ligands are illustrated on the right side.

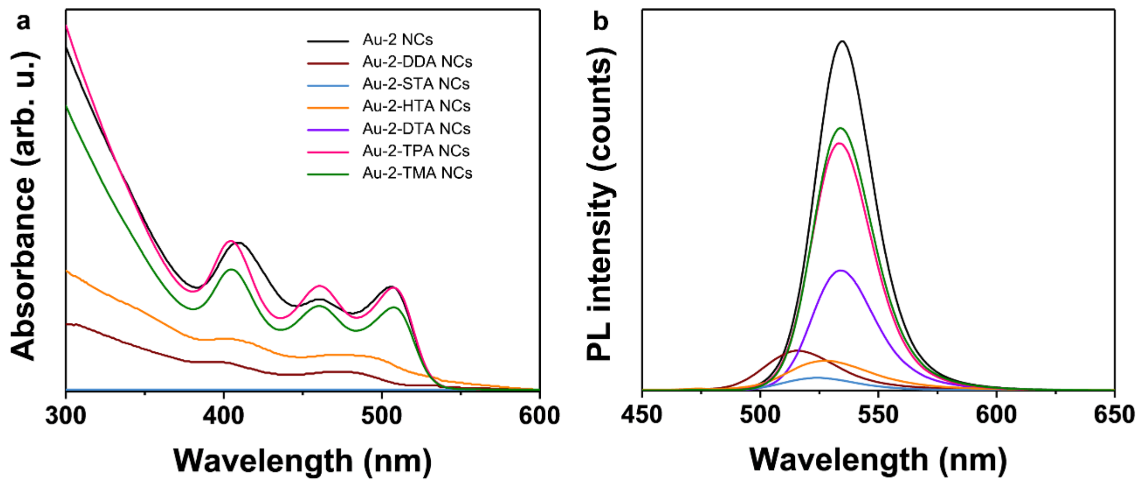

Supplementary Figure 50 Optical properties of Au-2 NCs added with different ligands. (a) UV-vis absorption and (b) PL spectra of Au-2, Au-2-DDA, Au-2-STA, Au-2-HTA, Au-2-DTA, Au-2-TPA, and Au-2-TMA NCs.

Supplementary Table 1. Fluorescent lifetime components of Au-1, Au-2, and Au-3 NCs obtained from TCSPC measurements. The proportion of each component is shown in brackets.

Gold NCs	τ_1 (ns)	τ_2 (ns)	$\tau_{ave.}$ (ns)	k_r (× 10^5 s^{-1})	k_{nr} (× 10^6 s^{-1})
Au-1	1.6 ± 0.01 (59.3%)	9.3 ± 0.09 (40.7%)	4.7 ± 0.07	4.3	212.3
Au-2	22.7 ± 0.51 (23.2%)	55.4 ± 0.38 (76.8%)	47.8 ± 0.38	10.7	10.2
Au-3	32.6 ± 0.71 (39.2%)	65.7 ± 1.03 (60.8%)	52.7 ± 0.92	15.3	3.7

Supplementary Table 2. Structural parameters extracted from the Au L_3-edge and $\chi(R)$ space spectra fitting of Au-1 NCs.

	Reduced Chi-square (χ_v^2)	R-factor (%)	amp/S_0^2	$N_{(Au\text{-}S\ path)}$	$R_{(Au\text{-}S\ path)}$ (Å)	$\sigma^2_{(Au\text{-}S\ path)}$ (10^{-3} Å^2)	ΔE_0 (eV)
Au-1	550.77	0.0551	0.83+/-0.14	1.2	2.333 ± 0.023	4.5+/-1.8	-3.14+/-1.95
			amp/S_0^2	$N_{(Au\text{-}Au\ path)}$	$R_{(Au\text{-}Au\ path)}$ (Å)	$\sigma^2_{(Au\text{-}Au\ path)}$ (10^{-3}Å^2)	ΔE_0 (eV)
			0.81+/-0.13	4.0	2.851 ± 0.028	5.6+/-2.7	-4.17+/-2.06

Supplementary Table 3. Structural parameters extracted from the Au L_3-edge and $\chi(R)$ space spectra fitting of Au-2 NCs.

	Reduced Chi-square (χ_v^2)	R-factor (%)	amp/S_0^2	$N_{(Au\text{-}S\ path)}$	$R_{(Au\text{-}S\ path)}$ (Å)	$\sigma^2_{(Au\text{-}S\ path)}$ (10^{-3}Å^2)	ΔE_0 (eV)
Au-2	562.37	0.0394	0.83+/-0.12	1.3	2.293 ± 0.011	3.8+/-1.4	2.77+/-0.98
			amp/S_0^2	$N_{(Au\text{-}Au\ path)}$	$R_{(Au\text{-}Au\ path)}$ (Å)	$\sigma^2_{(Au\text{-}Au\ path)}$ (10^{-3}Å^2)	ΔE_0 (eV)
			0.81+/-0.11	4.1	2.844 ± 0.015	5.8+/-2.5	4.04+/-2.12

Supplementary Table 4. Structural parameters extracted from the Au L_3-edge and $\chi(R)$ space spectra fitting of Au-3 NCs.

	Reduced Chi-square (χ_v^2)	R-factor (%)	amp/ S_0^2	$N_{(Au\text{-}S\ path)}$	$R_{(Au\text{-}S\ path)}$ (Å)	$\sigma^2_{(Au\text{-}S\ path)}$ (10^{-3}Å^2)	ΔE_0 (eV)
Au-3	511.27	0.0393	0.83+/-0.12	1.4	2.361 ± 0.031	3.9+/-1.6	3.84+/-1.36
			amp/ S_0^2	$N_{(Au\text{-}Au\ path)}$	$R_{(Au\text{-}Au\ path)}$ (Å)	$\sigma^2_{(Au\text{-}Au\ path)}$ (10^{-3}Å^2)	ΔE_0 (eV)
			0.81+/-0.11	4.1	2.836 ± 0.042	5.4+/-1.1	4.58+/-1.99

Supplementary Table 5. Time constants extracted from the global fitting of corresponding TA maps of Au-1, Au-2 and Au-3 NCs excited by different pump powers.

Pump power (μJ cm-2)	Au-1					Au-2					Au-3				
	τ_1 (fs)	τ_2 (ps)	τ_3 (ps)	τ_4 (ns)	Reduced χ^2	τ_1 (fs)	τ_2 (ps)	τ_3 (ps)	τ_4 (ns)	Reduced χ^2	τ_1 (fs)	τ_2 (ps)	τ_3 (ps)	τ_4 (ns)	Reduced χ^2
80	317 ±1.8	2.1 ±0.4	80.6 ±0.9	> 1	3.9×10^{-3}	240 ±1.2	2.9 ±0.3	80.7 ±0.6	> 1	3.1×10^{-3}	118 ±1.3	4.3 ±0.3	80.5 ±1.5	> 1	3.8×10^{-3}
120	320 ±1.8	2.2 ±0.3	80.6 ±1.2	> 1	5.2×10^{-3}	238 ±0.9	2.9 ±0.3	80.7 ±1.4	> 1	2.5×10^{-3}	115 ±1.3	4.2 ±0.2	80.5 ±1.5	> 1	2.6×10^{-3}
160	318 ±1.4	2.2 ±0.3	80.6 ±1.0	> 1	4.4×10^{-3}	239 ±1.1	2.8 ±0.2	80.7 ±1.5	> 1	4.5×10^{-3}	116 ±1.1	4.1 ±0.2	80.5 ±1.7	> 1	3.9×10^{-3}
240	319 ±1.7	2.1 ±0.2	80.6 ±1.4	> 1	5.7×10^{-3}	240 ±1.2	2.8 ±0.3	80.7 ±1.0	> 1	3.4×10^{-3}	117 ±0.9	4.2 ±0.1	80.5 ±1.6	> 1	3.3×10^{-3}
300	320 ±1.5	2.2 ±0.3	80.6 ±1.6	> 1	8.8×10^{-3}	240 ±1.2	2.9 ±0.2	80.7 ±1.3	> 1	4.1×10^{-3}	118 ±0.8	4.1 ±0.1	80.5 ±1.9	> 1	2.7×10^{-3}

Supplementary Table 6. Time constants extracted from the global fitting of corresponding TA maps of Au-1, Au-2, and Au-3 NCs excited by different pump energies.

Pump energy (eV)	Au-1					Au-2					Au-3				
	τ_1 (fs)	τ_2 (ps)	τ_3 (ps)	τ_4 (ns)	Reduced χ^2	τ_1 (fs)	τ_2 (ps)	τ_3 (ps)	τ_4 (ns)	Reduced χ^2	τ_1 (fs)	τ_2 (ps)	τ_3 (ps)	τ_4 (ns)	Reduced χ^2
3.10 (400 nm)	320 ±1.5	2.2 ±0.3	80.6 ±1.6	> 1	8.8×10^{-3}	240 ±1.2	2.9 ±0.2	80.7 ±1.3	> 1	4.1×10^{-3}	118 ±0.8	4.1 ±0.1	80.5 ±1.9	> 1	2.7×10^{-3}
2.88 (430 nm)	290 ±1.7	2.2 ±0.2	80.6 ±1.5	> 1	5.6×10^{-3}	200 ±1.4	3.0 ±0.4	80.7 ±1.5	> 1	4.5×10^{-3}	100 ±1.2	4.1 ±0.2	80.5 ±2.2	> 1	2.9×10^{-3}
2.70 (460 nm)	190 ±2.1	2.1 ±0.3	80.6 ±1.9	> 1	3.6×10^{-3}	170 ±2.0	3.0 ±0.4	80.7 ±1.6	> 1	1.2×10^{-3}	90 ±2.0	4.2 ±0.2	80.5 ±2.0	> 1	2.1×10^{-2}
2.53 (490 nm)		1.5 ±0.3	60.0 ±1.8	> 1	3.8×10^{-3}	100 ±1.8	2.4 ±0.3	80.5 ±1.4	> 1	2.3×10^{-3}	80 ±1.7	4.0 ±0.1	80.4 ±2.4	> 1	5.2×10^{-3}
2.48 (500 nm)							2.1 ±0.2	60.0 ±1.8	> 1	1.7×10^{-2}		3.3 ±0.2	64.0 ±2.2	> 1	3.5×10^{-3}
2.43 (510 nm)							1.9 ±0.2	56.0 ±1.6	> 1	3.0×10^{-3}		2.5 ±0.1	50.0 ±1.8	> 1	4.4×10^{-3}

Supplementary Table 7. Structural parameters extracted from the Au L_3-edge and $\chi(R)$ space spectra fitting of Au-2-TMPA NCs.

<table>
<tr><td rowspan="5">Au-2-TMPA</td><td>Reduced Chi-square (χ_v^2)</td><td>R-factor (%)</td><td>amp/ S_0^2</td><td>$N_{(Au\text{-}S\ path)}$</td><td>$R_{(Au\text{-}S\ path)}$ (Å)</td><td>$\sigma^2_{(Au\text{-}S\ path)}$ (10^{-3}Å^2)</td><td>ΔE_0 (eV)</td></tr>
<tr><td rowspan="4">562.37</td><td rowspan="4">0.0402</td><td>0.83+/-0.12</td><td>1.1</td><td>2.288 ± 0.011</td><td>4.4+/-1.9</td><td>2.78+/-1.06</td></tr>
<tr><td>amp/ S_0^2</td><td>$N_{(Au\text{-}Au\ path)}$</td><td>$R_{(Au\text{-}Au\ path)}$ (Å)</td><td>$\sigma^2_{(Au\text{-}Au\ path)}$ (10^{-3}Å^2)</td><td>ΔE_0 (eV)</td></tr>
<tr><td>0.81+/-0.11</td><td>4.3</td><td>2.814 ± 0.014</td><td>5.8+/-2.3</td><td>4.22+/-2.07</td></tr>
</table>

Supplementary Table 8. Structural parameters extracted from the Au L_3-edge and $\chi(R)$ space spectra fitting of Au-2-TMBA NCs.

	Reduced Chi-square (χ_v^2)	R-factor (%)	amp/ S_0^2	$N_{(Au\text{-}S\ path)}$	$R_{(Au\text{-}S\ path)}$ (Å)	$\sigma^2_{(Au\text{-}S\ path)}$ (10^{-3}Å^2)	ΔE_0 (eV)
Au-2-TMBA	603.02	0.0587	0.83+/-0.12	1.4	2.293 ± 0.012	4.3+/-1.3	2.81+/-1.01
			amp/ S_0^2	$N_{(Au\text{-}Au\ path)}$	$R_{(Au\text{-}Au\ path)}$ (Å)	$\sigma^2_{(Au\text{-}Au\ path)}$ (10^{-3}Å^2)	ΔE_0 (eV)
			0.81+/-0.11	3.8	2.821 ± 0.016	5.8+/-2.8	4.39+/-2.16
